# Glucocorticoids can induce BIM to trigger apoptosis in the absence of BAX and BAK1

**DOI:** 10.1038/s41419-020-2599-5

**Published:** 2020-06-08

**Authors:** Li Dong, David L. Vaux

**Affiliations:** 1 The Walter and Eliza Hall Institute of Medical Research, 1G Royal Parade, Parkville, VIC 3052 Australia; 2 Department of Medical Biology, The University of Melbourne, Parkville, VIC 3052 Australia

**Keywords:** Translation, Apoptosis

## Abstract

Cells from two murine lymphoid lines died 24–48 h after treatment with the glucocorticoid dexamethasone. Deletion of *Bax* and *Bak1* prevented rapid apoptosis, but treatment with dexamethasone for greater 6 days still led to cell death that was characterized by release of cytochrome c into the cytosol, activation of caspases, and loss of cell membrane integrity. In WEHI7 thymoma cells, this did not occur when *Bcl2l11* (*Bim)* was deleted in addition to *Bax* and *Bak1*. When these triple mutant lines were exposed to dexamethasone for 10 days, they arrested, but after dexamethasone was removed, they had 10-fold higher clone forming efficiency than *Bax*/*Bak1* double knock-out cells. Although induced over-expression of BIMs alone was not sufficient to induce the death of *Bax*^*−*/−^*Bak1*^*−*/−^*Bim*^*−*/−^ cells, they did die when BIMs was induced in the presence of dexamethasone. These results suggest that dexamethasone induces production of BIM together with other, as yet unidentified proteins, that cause release of cytochrome c and apoptosis in the absence of BAX and BAK1.

## Introduction

BAX and BAK1 are generally believed to be essential for cells to undergo apoptosis by the “intrinsic” or “mitochondrial” pathway^1^. Furthermore, BH3-only proteins such as BIM and BAD are thought to require presence of BAX or BAK1 in order to kill cells^2^. To test these beliefs, we carried out a series of experiments in WEHI7 thymoma cells, which can be induced to undergo apoptosis by the glucocorticoid dexamethasone (Dex)^3,4^.

In sensitive lymphoid cells such as WEHI7 cells, the glucocorticoid Dex induces apoptosis within 24–48 h^5,6^. When Dex-sensitive cell lines are transfected to over-express *Bcl2*^4^, or both *Bax* and *Bak1* are mutated in lymphoid cells^7^, they are much more resistant, indicating that the major way Dex induces rapid lymphocyte apoptosis is via activation of BAX and/or BAK1. These proteins cause cytochrome c to be released from the mitochondria into the cytosol^8^, where it binds to APAF1, activating the apoptosome and caspases^9^, so that cells lose plasma membrane integrity, as indicated by uptake up the dye propidium iodide (PI).

It has been well established that BAX and BAK1 can be activated, causing in increase in mitochondrial outer membrane permeability and release cytochrome c, when BH3-only proteins such as BCL2LII (BIM), PUMA, and BMF counter the anti-apoptotic activity of BCL2, BCLX, and MCL1^10^.

In thymocytes, it is clear that BIM plays a major role in triggering Dex-induced apoptosis, because thymocytes from *Bim* deleted mice are much more resistant to Dex than thymocytes from wild-type mice^6^.

In order to determine the requirements for pro- and anti-apoptotic BCL2 family members in Dex-induced apoptosis of cells of the murine WEHI7 thymoma line^3^, we determined the effect of mutating genes using CrispR/Cas9. We were surprised to find that although rapid Dex-induced apoptosis required BAX or BAK1, when *Bax*^−/−^*Bak1*^−/−^ WEHI7 cells were maintained in Dex for longer periods, they were still able to release cytochrome c from the mitochondria, activate the apoptosome, and undergo apoptosis. To confirm these unexpected results, we independently repeated them in another dexamethasone-sensitive lymphoid line that was initially derived from p53^−/−^ mice^11^.

## Results

**Fig. 1:**
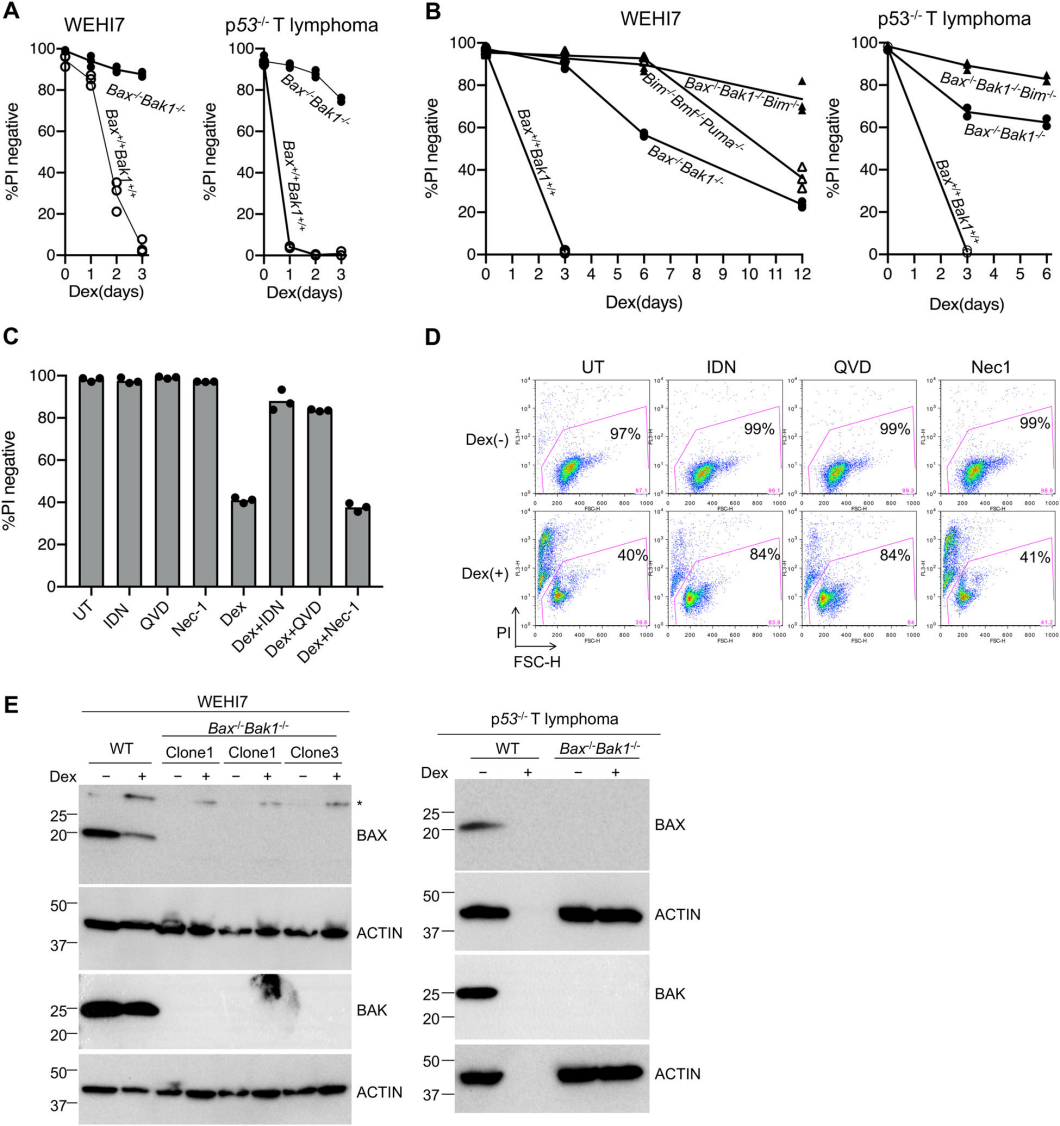
In the absence of BAX and BAK1, Dex can still cause cell death, but it takes much longer. **a** Independent *Bax*^*+/+*^*Bak1*^*+/+*^ (wild type; open circles) and *Bax*^−/−^*Bak1*^−/−^ (filled circles) WEHI7 lines (left), and *p53*^−/−^ T lymphoma cell lines (right), were treated with 1 µM Dex for 3 days, and viability was determined by propidium iodide (PI) uptake. Almost all of the wild-type cells died by day 3, whereas the lines lacking BAX and BAK1 remained PI negative. **b** WEHI7 cells (left) and *p53*^−/−^ T lymphoma cells (right) from each genotype (*Bax*^*+/+*^*Bak1*^*+/+*^, *Bax*^−/−^*Bak1*^−/−^*, Bim*^−/−^*Bmf*^−/−^*Puma*^−/−^, and *Bax*^−/−^*Bak1*^−/−^*Bim*^−/−^) were treated with 1 µM Dex for indicated times, and viability was determined by PI uptake. When cells were exposed to Dex for longer time periods, cells from *Bax*^−/−^*Bak1*^−/−^ lines also became PI positive. In comparison, fewer cells from independent lines lacking BAX, BAK1, and BIM (filled triangles), or lacking PUMA, BMF, and BIM (open triangles), died when exposed to Dex. Symbols represent independent clonal lines of each genotype. **c** Caspase inhibitors QVD or IDN, but not the RIPK1 inhibitor Nec-1, prevented PI uptake by Dex-treated *Bax*^−/−^*Bak1*^−/−^ WEHI7 cells at day 6. Independent *Bax*^−/−^*Bak1*^−/−^ WEHI7 lines were treated with 1 µM Dex and/or 10 µM QVD-OPh or 5 µM IDN-6556 or 50 µM Nec-1 for 6 days. Cells were harvested, resuspended in PBS containing PI and analyzed by flow cytometry. Data show means of two independent experiments each using three independent lines. **d** Dot plots of one of the *Bax*^−/−^*Bak1*^−/−^ WEHI7 independent clonal lines shown in **c**; numbers indicate the percent of PI-negative cells of a total of 10,000 cells analyzed per condition. **e** To confirm gene mutation in each of the clonal lines, whole-cell lysates from *Bax*^*+*/+^*Bak1*^*+*/+^ and *Bax*^−/−^*Bak1*^−/−^ WEHI7 cells (left panel) and *p53*^−/−^ T lymphoma cells (right panel) treated with 1 µM Dex for 24 h were subjected to western blot analysis. Note that the absence of protein in the Dex-treated WT *p53*^−/−^ T lymphoma cells is due to the death of most of the cells by 24 h.

Cells of the Dex sensitive, WEHI7 murine thymoma line, rapidly died by apoptosis after exposure to Dex, such that of >90% of cells were PI positive within 3 days (Fig. 1a open circles). The Dex-treated cells also expressed higher levels of *Bim* mRNA (RNAseq data not shown) and BIM protein, consistent with a model in which Dex causes the glucocorticoid receptor to bind DNA and induce expression of *Bim* mRNA, and the corresponding increase in BIM protein counters anti-apoptotic BCL2 family members to free BAX and BAK1 to activate, leading to release of cytochrome c from the mitochondria and cell death.

**Fig. 2:**
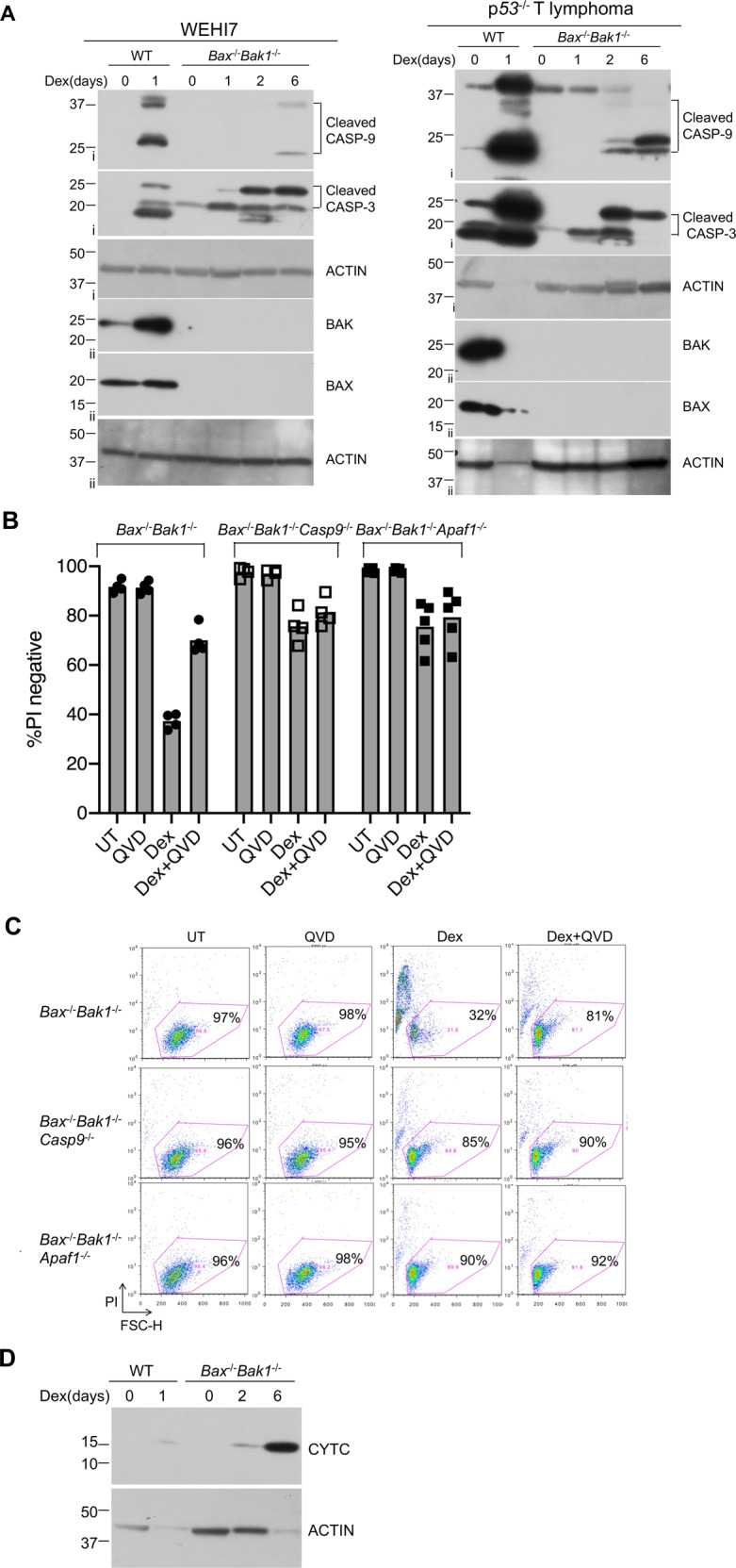
Dexamethasone can induce caspase activation in the absence of BAX and BAK1. **a** WEHI7 cells (left panel) and *p53*^*−/−*^ lymphoma cells (right panel) from each genotype (*Bax*^*+*^^/+^*Bak1*^*+*^^/+^ and *Bax*^−/−^*Bak1*^−/−^) were treated with 1 µM Dex for indicated times. Cell lysates were analyzed by western blot with antibodies to cleaved Caspase-3, cleaved Caspase-9, BAK, BAX, and ACTIN. Data show results of one of two independent experiments. Roman numerals to the left of blots (i–ii) indicate the membrane probed. **b** Mutating *Caspase-9* or *Apaf1* genes prevented Dex-induced PI uptake in *Bax*^−/−^*Bak1*^−/−^ WEHI7 clonal lines. In the lines mutant for *Caspase-9* or *Apaf1*, addition of QVD did not increase the percentage of PI-negative cells. Independent WEHI7 cell clones from each genotype (*Bax*^−/−^*Bak1*^−/−^, *Bax*^−/−^*Bak1*^−/−^*Casp9*^−/−^ and *Bax*^−/−^*Bak1*^−/−^*Apaf1*^*−/−*^) were treated with 1 µM Dex and/or 10 µM QVD for up to 6 days. Cells were harvested, resuspended in PBS containing PI, and analyzed by flow cytometry. Data show one of two independent experiments using four or five independent clonal lines. **c** Dot plots of independent clones from experiments shown in **b**; numbers represent the percent of PI-negative cells in a total of 10,000 cells analyzed per condition. **d** Dexamethasone induces cytochrome c release in a *Bax/Bak1* independent manner in WEHI7 cells. Cytoplasmic extracts from WT and *Bax*^*−/−*^*Bak1*^*−/−*^ WEH7 cells, which were treated with 1 µM DEX for 0 to 6 days, were subjected to western blot analysis, with antibody specific for cytochrome c (CYTC) and ACTIN. Results are from one of three independent experiments.

Unlike the parental cells, independent clones of WEHI7 cells in which genes for *Bax* and *Bak1* were mutated using CrispR/Cas9 (Fig. 1e) did not rapidly die in response to 1 µM Dex (Fig. 1a, filled circles). However, we found that after longer exposure to Dex, *Bax*^−/−^*Bak1*^−/−^ WEHI7 cells eventually did die, with 75% taking up propidium iodide (PI) by 12 days (Fig. 1b, left).

As we were surprised by these findings, and to ensure that they were not just a peculiarity of WEHI7 thymoma cells, we reproduced most of our experiments in an independent dexamethasone-sensitive lymphoid line^11^ derived from p53^−/−^ mice that was kindly provided to us by Andreas Strasser (Fig. 1a, right; 1b, right).

To determine whether the *Bax*^−/−^*Bak1*^−/−^ WEHI7 cells were dying by necroptosis, we added the RIPK1 inhibitor necrostatin (Nec-1)^12^ to the culture medium, but it did not affect the number of cells that died (Fig. 1c, d). To see whether caspases were involved in their death, we included the caspase inhibitors QVD-OPh (QVD) or IDN-6556 (IDN). As these caspase inhibitors almost completely prevented PI uptake (Fig. 1c, d), we concluded that treatment with Dex led to caspase activation, even in the *Bax*^−/−^*Bak1*^−/−^ WEHI7 cells.

To determine which caspases became processed when the *Bax*^−/−^*Bak1*^−/−^ WEHI7 cells were treated with Dex, we analyzed lysates by western blot using antibodies specific for cleaved caspases. As shown in Fig. 2a, by 24 hrs Dex-induced cleavage and activation of both caspase 9 and caspase 3 in wild-type WEHI7 cells. Although no activated caspases could be detected by this time in the *Bax*^−/−^*Bak1*^−/−^ WEHI7 cells, after 6 days exposure to Dex, both caspase 9 and caspase 3 were cleaved. Similarly, cleaved caspases could be detected in the *Bax*^−/−^*Bak1*^−/−^ p53^−/−^ T lymphoma line after 2 days treatment with Dex (Fig. 2a).

**Fig. 3:**
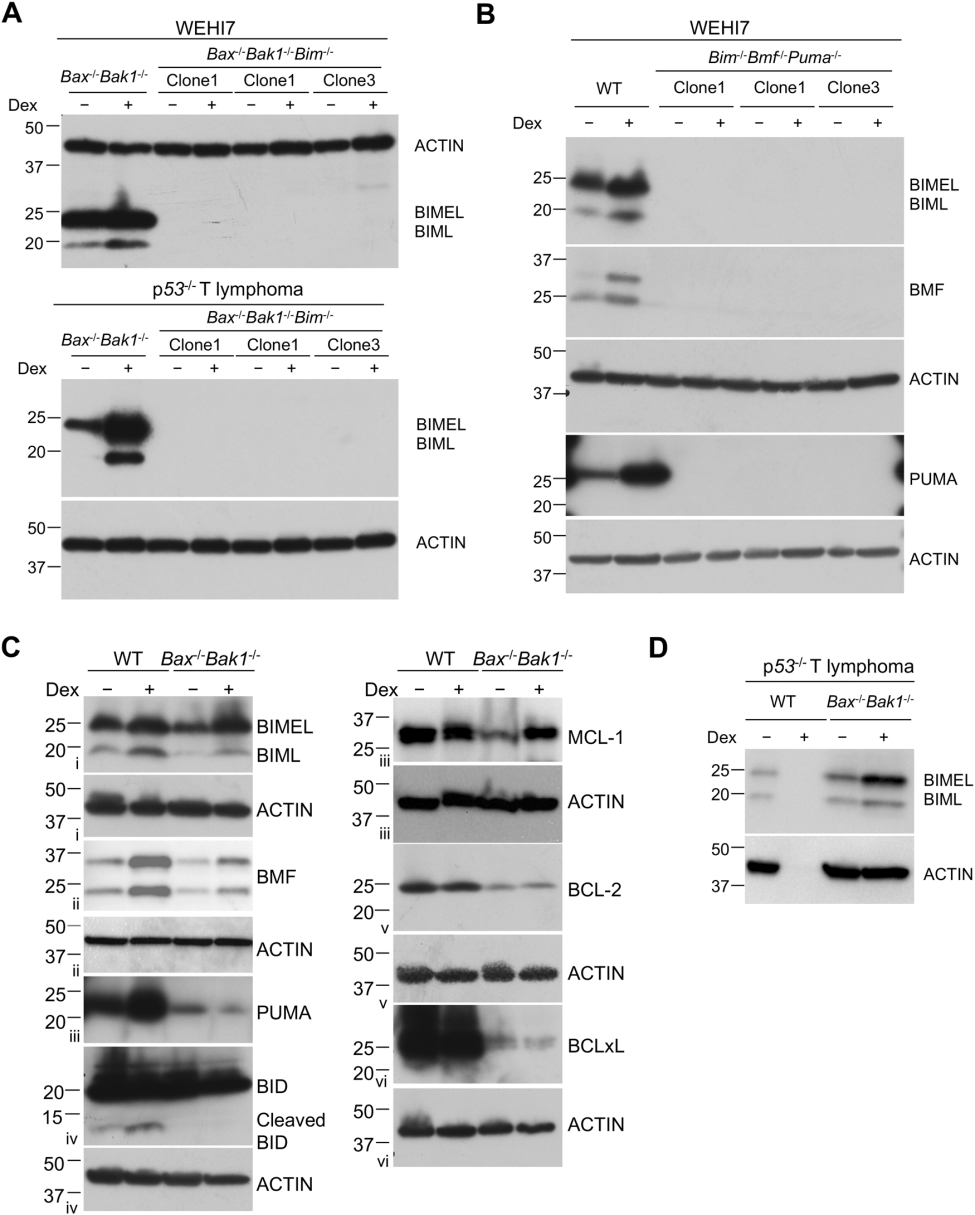
Characterization of clonal lymphoid lines mutant for combinations of pro-apoptotic BCL2 family proteins. **a** Whole-cell lysates from *Bax*^*−/−*^*Bak1*^*−/−*^ and three independent *Bax*^*−/−*^*Bak1*^*−/−*^*Bim*^*−/−*^ cell clones treated with 1 µM Dex treatment for 24 hrs were subjected to western blot analysis to detect BIM protein. Upper panel: WEHI7 mutant lines; lower panel: *p53*^*−/−*^ T lymphoma mutant lines. **b** *Bax*^*+/+*^*Bak1*^*+/+*^ WEHI7 cells expressing Cas9 were transduced with sgRNAs targeting mouse *Bim*, *Bmf* and *Puma*. Following treatment with doxycycline to induce sgRNA expression, clones were isolated and validated for absence of BIM, BMF, and PUMA by western blotting after 24 hrs treatment with 1 µM Dex. **c** Wild type (WT) and *Bax*^−/−^*Bak1*^−/−^ WEHI7 cells were treated for 24 hrs with 1 µM Dex and lysates were run on replicate gels and analyzed by western blot using antibodies specific for the indicated BCL2 family proteins. Roman numerals to the left of blots (i–vi) indicate the membranes probed. **d** Whole-cell lysates from *Bax*^+/+^*Bak1*^+/+^ and *Bax*^−/−^*Bak1*^−/−^ *p53*^−/−^ T lymphoma cells treated with 1 µM Dex for 24 hrs were tested by western for expression of BIM. Note that the absence of protein in the Dex-treated WT *p53*^−/−^ T lymphoma cells is due to the death of most of the cells by 24 hours.

Caspase 9 and 3 can be activated by APAF1 in the apoptosome^9^. To determine whether APAF1 was required for Dex-induced death of the *Bax*^−/−^*Bak1*^−/−^ WEHI7 cells, we mutated the genes for APAF1 to generate multiple, independent, *Bax*^−/−^*Bak1*^−/−^*Apaf1*^−/−^ WEHI7 clones, treated them with Dex, and assessed their viability by measuring PI uptake. In these clones, and in *Bax*^−/−^*Bak1*^−/−^*Casp9*^−/−^ WEHI7 clones, Dex caused much less PI uptake than in the *Bax*^−/−^*Bak1*^−/−^ WEHI7 cells (Fig. 2b, c and Supplementary Fig. S1). Furthermore, although the caspase inhibitor QVD was able to protect the *Bax*^−/−^*Bak1*^−/−^ WEHI7 cells, it had no impact on the amount of PI uptake in the *Bax*^−/−^*Bak1*^−/−^*Apaf1*^−/−^ and *Bax*^−/−^*Bak1*^−/−^*Casp9*^−/−^ WEHI7 clones (Fig. 2b, c). We infer from these experiments that in *Bax*^−/−^*Bak1*^−/−^ WEHI7 cells, long-term treatment with Dex can lead to activation of APAF1 and the apoptosome, leading to processing and activation of caspases, and culminating in loss of plasma membrane integrity and uptake of PI.

APAF1 is activated when it is bound by cytochrome c (Cytc), which is released from the mitochondria into the cytosol during apoptosis^13^. To see whether release of Cytc was triggered by long-term Dex treatment of the *Bax*^−/−^*Bak1*^−/−^ WEHI7 cells, we treated the cells will Dex for 0 to 6 days, and looked at cytosol fractions, looking for appearance of Cytc in the cytoplasm. As shown in Fig. 2d and Supplementary Fig. S2a, after 6 days of treatment with Dex, Cytc could readily be detected in the cytosol. Although release of Cytc from the mitochondria (and death of the cells) was much slower than in the wild-type WEHI7 cells, Dex was still able to cause the release of Cytc, in the absence of BAX and BAK1.

**Fig. 4:**
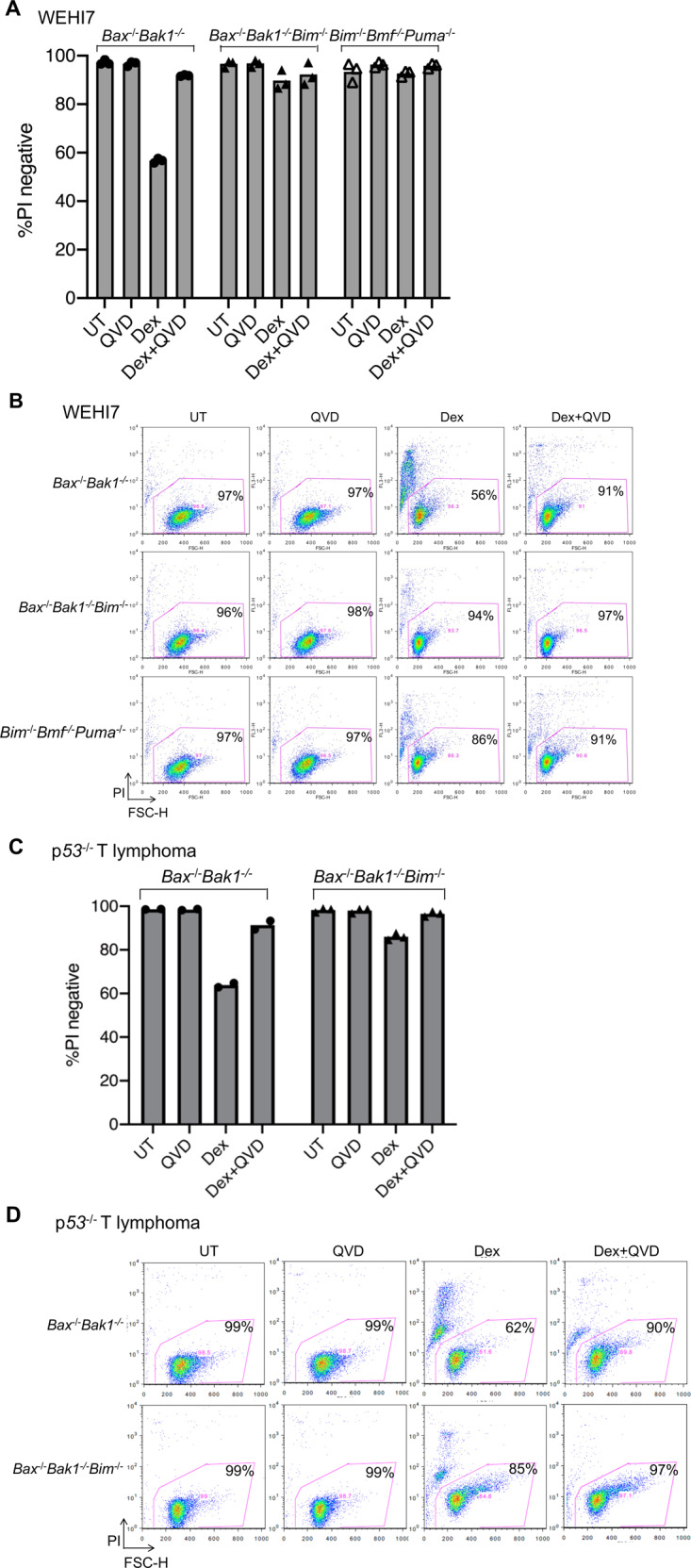
In the absence of BIM, the ability of Dex to cause death of Bax^−/−^Bak1^−/−^, and Bmf^−/−^Puma^−/−^ WEHI7 cells was reduced. **a** Multiple independent clonal *Bax*^−/−^*Bak1*^−/−^, *Bim*^−/−^*Bmf*^−/−^*Puma*^−/−^ and *Bax*^−/−^*Bak1*^−/−^*Bim*^−/−^ WEHI7 lines were treated with 1 µM Dex and/or 10 µM QVD-OPh for 6 days. Cells were analyzed by flow cytometry for uptake of PI. Each symbol represents an independently derived clonal line. **b** Dot plots show independent clones from one of the three independent experiments shown in **a**. Numbers indicate the percent of PI-negative cells in a total of 10,000 cells analyzed per condition. **c** In two independent experiments, *Bax*^*−/−*^*Bak1*^*−/−*^ parental, and three independent *Bax*^−/−^*Bak1*^−/−^*Bim*^−/−^ mutant *p53*^−/−^ T lymphoma cell lines were treated with 1 µM Dex and/or 10 µM QVD-OPh for 6 days. Cells were harvested, resuspended in PBS containing PI and analyzed by flow cytometry. **d** Dot plots show the parental *Bax*^*−/−*^*Bak1*^*−/−*^ *p53*^−/−^ T lymphoma cell line and one of the three *Bax*^−/−^*Bak1*^−/−^*Bim*^−/−^ mutant *p53*^−/−^ T lymphoma cell lines shown in **c**. Numbers indicate the percentage of PI-negative cells from a total of 10,000 cells analyzed per condition.

Western blots were performed to determine which genes were induced by treatment with Dex. After 24 hours treatment, we saw increased levels of BIM in both in the WEHI7 and p53^−/−^ T lymphoma lines (Fig. 3c, d), and the WEHI7 cells also increased BMF (Fig. 3c). As BIM was induced by Dex, and has previously been shown to play a major role in apoptosis of lymphoid cells treated with dexamethasone^6,14^, we generated multiple independent clones of *Bax*^−/−^*Bak1*^−/−^*Bim*^−/−^ WEHI7 cells, to see if they would die in response to Dex treatment (Fig. 3a). As shown in Fig. 4a, b, while 6 days treatment with Dex caused around half of the *Bax*^−/−^*Bak1*^−/−^ WEHI7 cells to become PI positive, and this could be blocked with the caspase inhibitor QVD, less than 15% of the *Bax*^−/−^*Bak1*^−/−^*Bim*^−/−^ WEHI7 clones became PI positive, and this was unaffected by QVD treatment. This indicates BIM is necessary for Dex to cause caspase activation in the *Bax*^−/−^*Bak1*^−/−^ WEHI7 cells.

As with the WEHI7 lines, mutation of BIM in addition to BAX and BAK1 in the p53^−/−^ T lymphoma cells also reduced the percentage of cells that took up PI following Dex treatment, although some *Bax*^−/−^*Bak1*^−/−^*Bim*^−/−^ cells did take up PI, and this was reduced by treating the cells with QVD (Figs. 3a and 4c, d). This suggests that while Dex-induced BIM can cause death of p53^−/−^ T lymphoma cells in the absence of BAX and BAK1, there are proteins in addition to BIM that can lead to caspase activation and PI uptake independently of BAX, BAK1, and BIM.

**Fig. 5:**
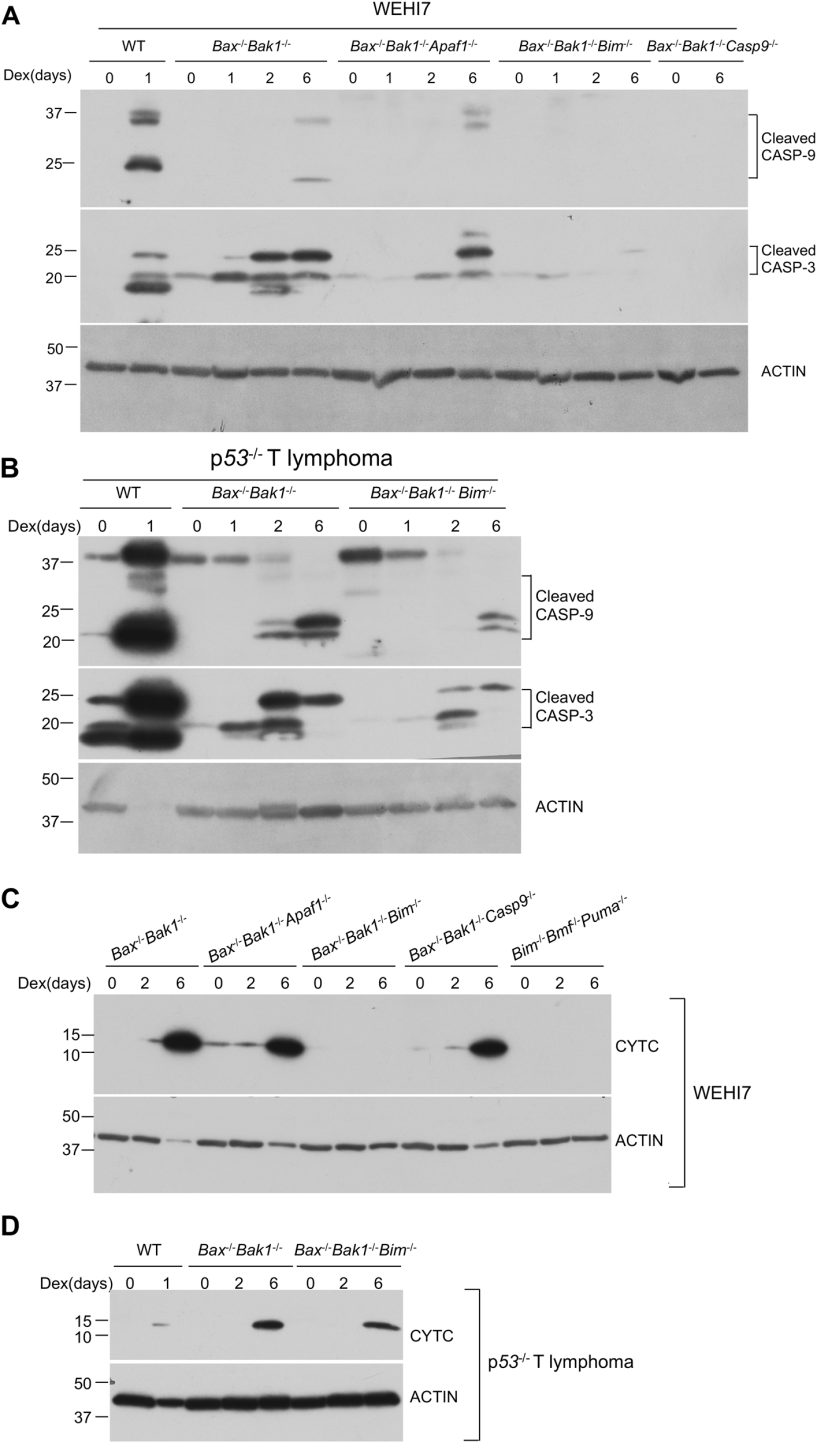
Dex-induced caspase activation and cytochrome c release in the absence of BAX and BAK1, but this failed to occur if BIM was also deleted. **a** *Bax*^*+*/+^*Bak1*^*+*/+^ (WT), *Bax*^−/−^*Bak1*^−/−^, *Bax*^−/−^*Bak1*^−/−^*Casp9*^−/−^, *Bax*^−/−^*Bak1*^−/−^*Apaf1*^*−/−*^, *Bim*^−/−^*Bmf*^−/−^*Puma*^−/−^ and *Bax*^−/−^*Bak1*^−/−^*Bim*^−/−^ clonal WEHI7 lines were treated with 1 µM Dex for indicated times. Cell lysates were analyzed by western blot using antibodies specific for cleaved Caspase-3, cleaved Caspase-9, and ACTIN. Note, the first 6 lanes of these blots are also shown in left panel of Fig. 2a. **b** *Bax*^*−/−*^*Bak1*^*−/−*^ and *Bax*^−/−^*Bak1*^−/−^*Bim*^−/−^ *p53*^*−/−*^ T lymphoma lines were treated with 1 µM Dex for indicated times. Whole-cell lysates were analyzed by western blot using antibodies specific for cleaved Caspase-3, cleaved Caspase-9, and ACTIN. Note, the first 6 lanes of these blots are also shown in right panel of Fig. 2a. **c** *Bax*^*+*/+^*Bak1*^*+*/+^, *Bax*^−/−^*Bak1*^−/−^, *Bax*^−/−^*Bak1*^−/−^*Casp9*^−/−^, *Bax*^−/−^*Bak1*^−/−^*Apaf1*^*−/−*^, *Bim*^−/−^*Bmf*^−/−^*Puma*^−/−^ and *Bax*^−/−^*Bak1*^−/−^*Bim*^−/−^ WEHI7 lines were treated with 1 µM Dex for indicated times. Cytoplasmic extracted fractions were subjected to western blot analysis, using antibodies specific for cytochrome c (CYTC), and ACTIN. **d** *Bax*^*−/−*^*Bak1*^*−/−*^ and *Bax*^−/−^*Bak1*^−/−^*Bim*^−/−^ *p53*^−/−^ T lymphoma lines were treated with 1 µM Dex for indicated times. Cytoplasmic fractions were subjected to western blot analysis, using antibodies specific for CYTC, and ACTIN.

Moreover, as shown in Fig. 5a, Dex-induced cleavage and activation of both caspase 9 and caspase 3 in the *Bax*^−/−^*Bak1*^−/−^ WEHI7 clones after 6 days treatment, but not in *Bax*^−/−^*Bak1*^−/−^ *Bim*^−/−^ or *Bax*^−/−^*Bak1*^−/−^ *Casp9*^−/−^ WEHI7 clones, and the pattern of caspase cleavage was altered and incomplete in the *Bax*^−/−^*Bak1*^−/−^ *Apaf1*^−/−^ WEHI7 clones. Cleavage of both caspase 9 and caspase 3 in Dex-treated *Bax*^−/−^*Bak1*^−/−^*Bim*^−/−^ *p53*^−/−^ T lymphoma cells was much less than in the parental *Bax*^−/−^*Bak1*^−/−^ *p53*^−/−^ T lymphoma cells (Fig. 5b).

Furthermore, as shown in Fig. 5c and Supplementary Fig. S2b, after 6 days treatment with Dex, Cytc was released into the cytosol of *Bax*^−/−^*Bak1*^−/−^ WEHI7 clones, and also of *Bax*^−/−^*Bak1*^−/−^*Apaf1*^−/−^ and *Bax*^−/−^*Bak1*^−/−^*Casp9*^−/−^ WEHI7 clones, but it was not released from cells that not only lacked BAX and BAK1, but also lacked BIM. These experiments show that not only can Dex cause Cytc to be released independently of BAX and BAK1, BIM is required for this to occur. BIM is allowing Cytc to be released from the mitochondria independently of BAX and BAK1.

Consistent with the partial protection of the *Bax*^−/−^*Bak1*^−/−^*p53*^−/−^ T lymphoma cells afforded by additionally mutating *Bim*, in the *Bax*^−/−^*Bak1*^−/−^*Bim*^−/−^ *p53*^−/−^ T lymphoma cells release of Cytc into the cytosol was reduced, but not eliminated (Fig. 5d and Supplementary Fig. S2c). Therefore, in the p53^−/−^ T lymphoma line, as in the WEHI7 cells, Dex could induce cytochrome c release and activation of the apoptosome in the absence of BAX and BAK1.

WEHI7 clones mutant for *Puma*, *Bmf*, and *Bim*, but with wild-type *Bax* and *Bak1*, responded the same way to Dex treatment as the *Bax*^−/−^*Bak1*^−/−^*Bim*^−/−^ WEHI7 clones (Figs. 3b and 4a, b), in that less than 15% became PI positive after 6 days treatment with Dex, and addition of QVD gave no additional protection. Consistent with this, Cytc was not released when Dex was used to treat *Bim*^−/−^*Bmf*^−/−^*Puma*^−/−^ WEHI7 clones (Fig. 5c). Therefore, for Dex to induce apoptosis of WEHI7 cells, they need to have one or more of the BH3 only proteins PUMA, BMF and BIM. If BAX or BAK1 is present, the cells undergo apoptosis rapidly, but in the absence of BAX and BAK1, BIM is still capable of inducing Cytc release and apoptosis, but at a much slower rate.

**Fig. 6:**
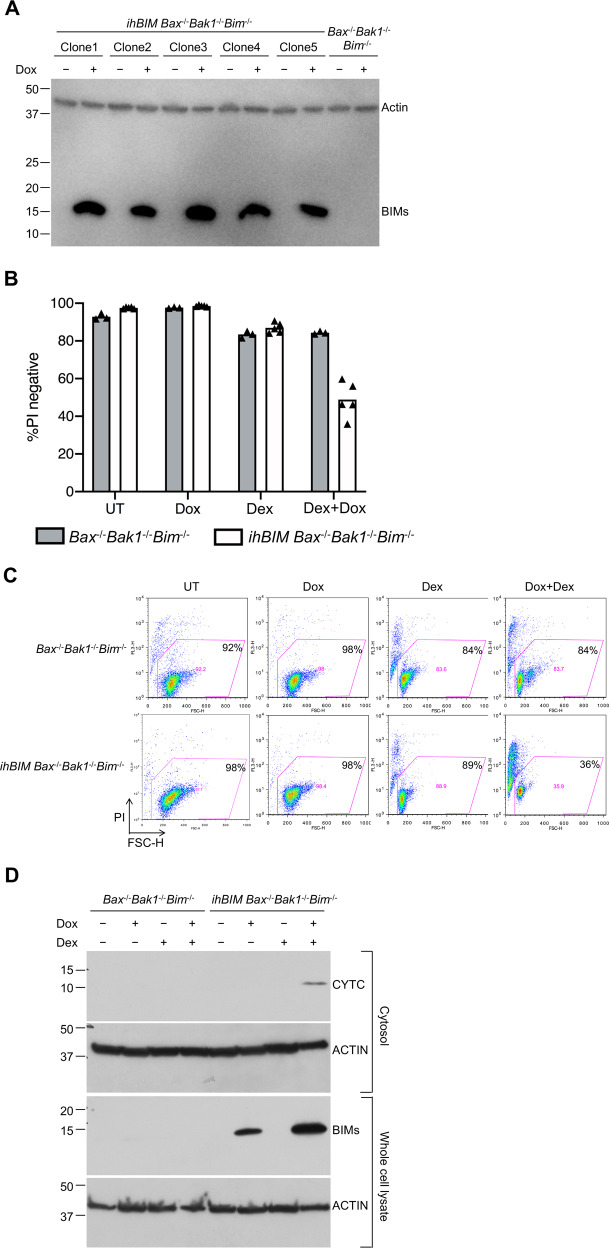
Induced expression of hBIMs was not sufficient to death of untreated cells, but did restore sensitivity of Bax^−/−^Bak1^−/−^Bim^−/−^ WEHI7 cells to treatment with dexamethasone. **a** *Bax*^−/−^*Bak1*^−/−^*Bim*^*−*/−^ WEHI7 cells were transduced with a lentiviral construct expressing human BIMs from a doxycycline-inducible promoter. Five independent *ihBIM Bax*^−/−^*Bak1*^−/−^*Bim*^*−*/−^ WEHI7 clonal cell lines were cultured for 3 days in the presence or absence of 1 µg/ml doxycycline (Dox). Bim expression was monitored by immunoblotting, using ACTIN as loading control. **b** *Bax*^−/−^*Bak1*^−/−^*Bim*^−/−^ parental cells (gray bars) and five independent *Bax*^−/−^*Bak1*^−/−^*Bim*^−/−^ inducible *hBIMs* (*ihBIM*) WEHI7 cell clones (white bars) were cultured for 6 days in the presence or absence of 1 µg/ml Dox and/or 1 µM Dex. Cell death was assessed by PI uptake using flow cytometry. For cell death to occur, both treatment with Dex, as well as induction of BIMs, were necessary. **c** Dot plots show one of the five independent clones as shown in **b**, numbers represent the percent of PI-negative cells of a total of 10,000 cells analyzed per condition. **d** *Bax*^−/−^*Bak1*^−/−^*Bim*^−/−^ WEHI7 cells and those from *ihBIM Bax*^−/−^*Bak1*^−/−^*Bim*^−/−^ WEHI7 lines were cultured for 6 days in the presence or absence of 1 µg/ml Dox and/or 1 µM Dex. Cytoplasmic extracts were subjected to western blot analysis, with antibodies specific for CYTC and ACTIN. For CYTC to be released into the cytosol, both treatment with Dex, as well as induction of BIMs, were necessary.

To determine whether expression of BIM was sufficient to trigger apoptosis in the absence of BAX and BAK1, we inserted a cDNA for human BIMs into a doxycycline (Dox)-inducible lentiviral vector, and used it to generate independent stable cell lines in which hBIMs could be induced by addition of Dox (Fig. 6a). As shown in Fig. 6b, induction of hBIMs by Dox for 6 days did not cause cells from *ihBIM Bax*^−/−^*Bak1*^−/−^*Bim*^−/−^ WEHI7 clones to die. Addition of Dex alone caused the cells to shrink, but also did not cause the cells to die, which is consistent with previous reports that Bim expression alone was not sufficient to kill cells in the absence of BAX and BAK1^2^. However, when cells were treated with Dox to induce hBIMs, and also treated with Dex, about 50% of the cells died as indicated by uptake of PI (Fig. 6b, c).

While induction of hBIMs alone did not cause Cytc to be released into the cytoplasm in cells lacking BAX and BAK1, and there was no detectable release when these cells were treated just with Dex, when hBIMs was induced by Dox in cells also treated with Dex for 6 days, Cytc was released from the mitochondria into the cytosol (Fig. 6d). We believe the Cytc detected in the cytosolic fractions was not due to mitochondrial contamination, because the abundant mitochondrial protein voltage-dependent anion channel 1 (VDAC-1) could not be detected in these fractions (as shown in supplementary Fig. S2d). Altogether, these experiments show that in Dex-treated cells that lack BAX and BAK1, BIM is necessary and sufficient to cause Cytc release and apoptosis. These results also imply that in addition to inducing BIM, Dex induces other protein(s) that are required for BAX/BAK1 independent release of Cytc.

While activation on the apoptosome is necessary for processing of caspase 9, caspase 3, and rapid cell death as indicated by uptake of PI, the point of no return leading to cell death—as indicated by loss of clonagenic capacity—occurs upstream of the apoptosome, prior to, or at the point of, mitochondrial outer membrane permeability and release of cytochrome c^15^. To determine the point at which cells were committed to die, we exposed wild type and mutant cells to dexamethasone, then washed the cells, and cultured them in soft agar to determine their clonagenic potential.

**Fig. 7:**
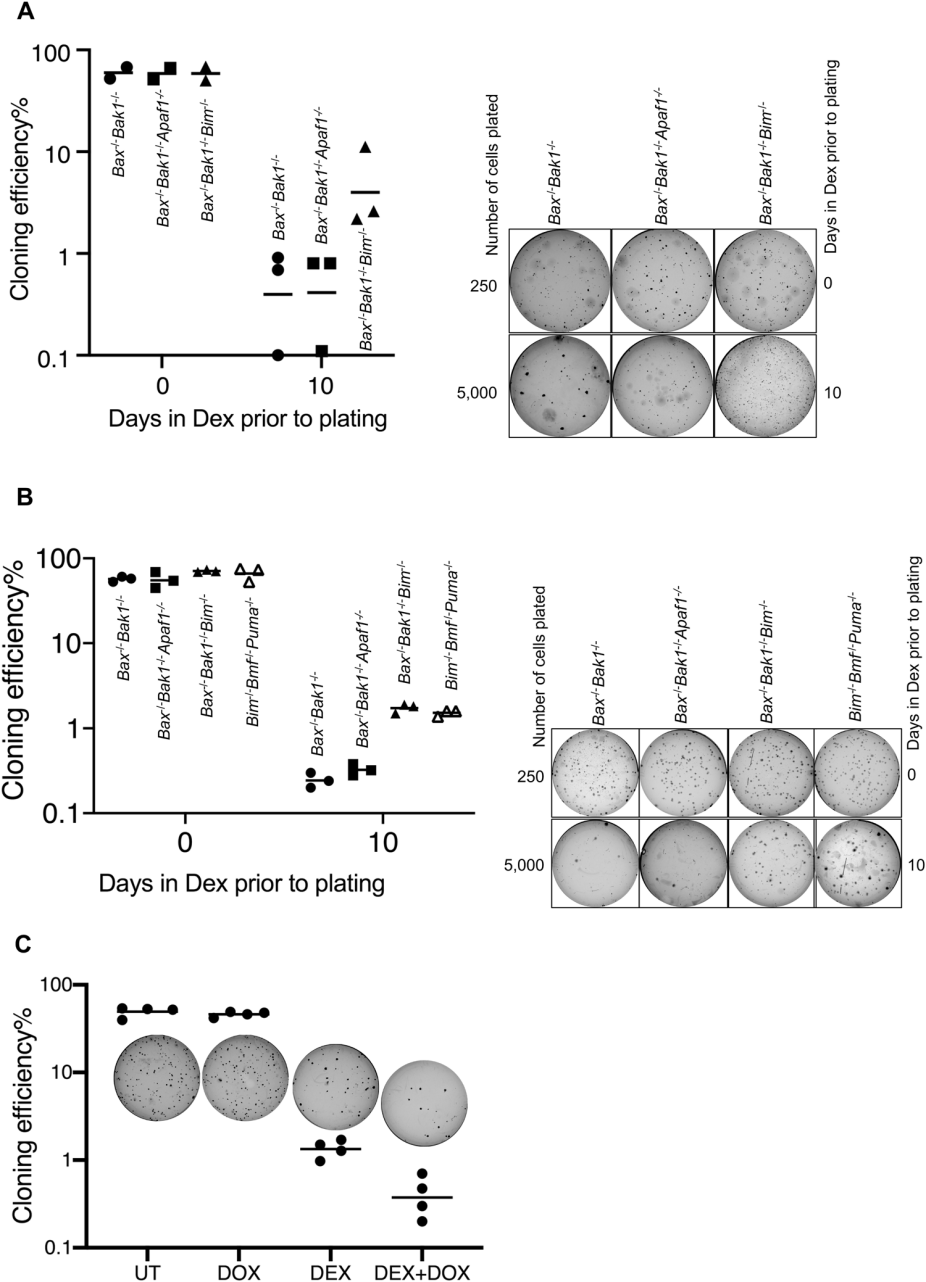
Deletion of BIM increased clonogenic survival of WEHI7 cells in response to Dex. **a** One representative WEHI7-derived clone of each genotype (*Bax*^*−/−*^*Bak1*^*−/−*^, *Bax*^*−/−*^*Bak1*^*−/−*^*Apaf1*^*−/−*^ and *Bax*^*−/−*^*Bak1*^*−/−*^*Bim*^*−/−*^) was cultured for 10 days in the presence or absence of 1 µM Dex. Cells were then washed, and plated in soft agar without Dex at a density of 5000 cells per well. Cells without Dex pre-treatment were plated at a lower density of 250 cells per well to generate countable numbers of colonies. After 14 days, the cloning efficiency was calculated by dividing the number of colonies by the number of cells initially plated in each well, and expressed as percentages. The results are from three independently conducted experiments. Photos of one of the three experiments are shown on the right. **b** Three independent WEHI7 cell clones from each genotype (*Bax*^*−/−*^*Bak1*^*−/−*^, *Bim*^*−/−*^*Bmf*^*−/−*^*Puma*^*−/−*^*Bax*^*−/−*^*Bak1*^*−/−*^*Apaf1*^*−/−*^ and *Bax*^*−/−*^*Bak1*^*−/−*^*Bim*^*−/−*^) were cultured for 10 days in the presence or absence of 1 µM Dex. Cells were then washed free of Dex, and plated in soft-agar medium for 14 days. Photos of one of the three experiments are shown on the right. **c** Four independent *ihBIM Bax*^*−/−*^*Bak1*^*−/−*^*Bim*^*−/−*^ WEHI7 cell clones were cultured for 10 days in the presence of 1 µM Dex and/or 1 µg/ml Dox. Cells were then washed free of Dex, and plated in soft-agar medium at a density of 4000 cells per well. Cells without Dex pre-treatment were plated at a lower density of 400 cells per well. Colonies were counted 14 days after plating.

After 10 days exposure to Dex, the clone forming potential of cells from independent *Bax*^−/−^*Bak1*^−/−^WEHI7 lines was reduced to 0.4% (Fig. 7a, b). *Bax*^−/−^*Bak1*^−/−^*Apaf1*^−/−^ WEHI7 cells had a similarly low clone forming capacity, even though fewer of these cells took up PI in short-term cell death assays (Fig. 2b). This is consistent with a requirement for APAF1 to induce caspase activation and rapid loss of plasma membrane integrity, but not being required for cells to ultimately die^15^. In contrast, *Bax*^−/−^*Bak1*^−/−^*Bim*^−/−^ WEHI7 lines and *Bim*^−/−^*Bmf*^−/−^*Puma*^−/−^ WEHI7 lines maintained ~10-fold higher clonagenic capacity. Notably, when hBIMs was induced by Dox in ihBIM *Bax*^*−/−*^*Bak1*^*−/*^^−^*Bim*^*−/−*^ WEHI7 cells treated with Dex for 10 days, the clonagenic capacity was only about 30% of that of cells treated only with Dex (Fig. 7c). These data showed that presence of BIM could reduce the long-term clonagenic capacity survival of WEHI7 lines, even in the absence of BAX and BAK1.

These experiments suggest that in some Dex-treated cells, BIM can act in the absence of BAX and BAK1 to cause cell death, but requires the presence of one or more other Dex-induced proteins. Of course, we wondered how BIM was able to cause Cytc release, and what these proteins might be. We hypothesized that the protein induced by Dex that allows BIM to cause Cytc release in the absence of BAX and BAK1 would be a BIM-binding protein that could act like BAX and BAK1 to form pores in the outer mitochondrial membrane.

**Fig. 8:**
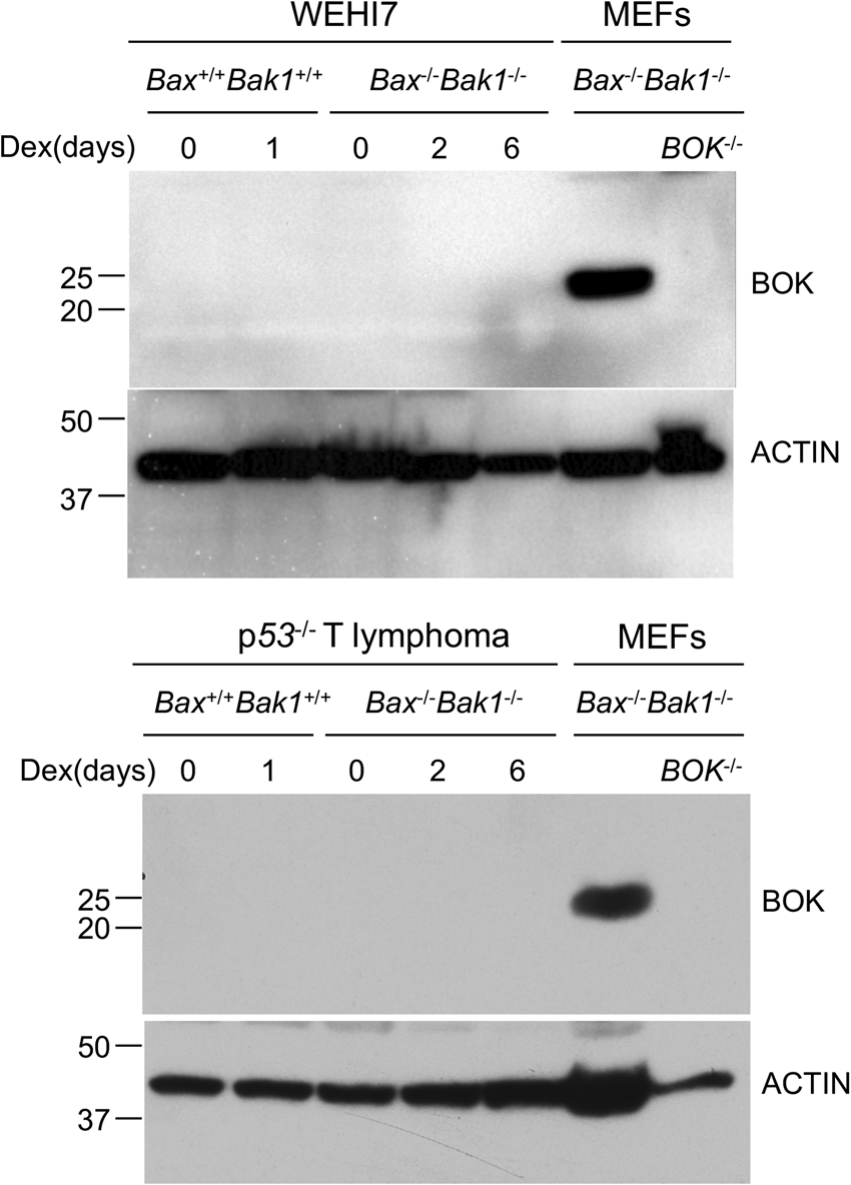
BOK is undetectable in WEHI7 and p53^−/−^ lymphoma cells. Whole-cell lysis of *Bax*^*−/−*^*Bak1*^*−/−*^ and *Bax*^−/−^*Bak1*^−/−^ WEH7 cells (upper panel) and p53^−/−^ T lymphoma cells (lower panel), were treated with 1 µM Dex for the indicated times and analyzed by western blot. Membranes were incubated with a rabbit anti–BOK polyclonal antibody and subsequently with a monoclonal antibody to ACTIN. Lysates from *Bax*^*−/−*^*Bak1*^*−/−*^ and *Bax*^−/−^*Bak1*^−/−^ *Bok*^−/−^ mouse embryonic fibroblast lines (MEFs) were used as positive and negative controls. The results of one of two independent experiments are shown.

As BOK is a BAX/BAK1-like pro-apoptotic protein^16^, we looked for BOK mRNA and protein, but we could not detect it in the WEHI7 or the p53 T lymphoma cells whether they were treated with Dex or not (Fig. 8 and RNAseq data not shown).

To further exclude the possibility that the BAX-like BH3-only protein BID may play a role in Dex-induced killing in the absence of BAX and BAK1, we mutated BID in the *Bax*^−/−^*Bak1*^−/−^ WEHI7 cells. Removing BID had no significant impact on the percentage of PI-positive cells that were induced by Dex treatment of *Bax*^−/−^*Bak1*^−/−^ WEHI7 lines (Supplementary Fig. S6).

Because opening of a cyclosporine A (CsA)-sensitive mitochondrial permeability transition pore (mPTP) has been implicated in apoptosis^17^, we investigated whether CsA could inhibit death of *Bax*^−/−^*Bak1*^−/−^ WEHI7 cells treated with Dex. We found that CsA had no influence on Dex-induced apoptosis of *Bax*^−/−^*Bak1*^−/−^ WEHI7 cells (Supplementary Fig. S10).

Although they inhibit apoptosis, the anti-apoptotic proteins BCL2, BCLXL and MCL-1 have similar folds to BAX and BAK1. We hypothesized that they might also have a latent pro-apoptotic activity that could be revealed by BIM. To test this, we mutated genes for all three proteins, individually and in combination, in the *Bax*^−/−^*Bak1*^−/−^ WEHI7 cells. As shown in Supplementary Figs. S3, S4, S5, S8), we saw no difference in the sensitivity of these cells to Dex, compared to the parental *Bax*^−/−^*Bak1*^−/−^ WEHI7 cells. In addition, addition of the BCL2-specific inhibitor ABT199 or the BCL2/BCLXL dual inhibitor ABT737 had no influence on the Dex-induced killing of *Bax*^−/−^*Bak1*^−/−^ WEHI7 cells (Supplementary Fig. S9). These experiments show that in the absence of BAX and BAK1, BCL2, BCLXL and MCL-1 do not affect survival of the Dex-treated WEHI7 cells.

Previous studies have suggested that VDAC proteins, in particular VDAC2, are involved in the release of cytochrome c across the mitochondrial outer membrane^18,19^. We therefore mutated VDAC2 in the *Bax*^−/−^*Bak1*^−/−^ WEHI7 cells. As shown in Supplementary Fig. S7, the absence of VDAC2 had no impact on Dex-induced killing of *Bax*^−/−^*Bak1*^−/−^ WEHI7 cells.

## Discussion

We began these experiments to extend the observations of Flomerfelt and Miesfeld who used mutagenesis and complementation assays to look for genes necessary for WEHI7 murine thymoma cells to die when exposed to dexamethasone (Dex)^4^. Flomerfelt and Miesfeld concluded that when the glucocorticoid receptors are bound by Dex, they activate a cell death pathway that could be blocked by over-expression of BCL2. In addition to genes for the glucocorticoid receptor, this pathway required 5 or 6 unknown gene products that were each required to be present prior to the point at which BCL2 functioned. However, we were not able to identify any of these genes, and were surprized to find that cells with mutations to both BAX and BAK1 could still be induced to undergo apoptosis if exposed to Dex for more than a few days.

We found that activation of the glucocorticoid receptors by dexamethasone induces expression of BIM, and together with other induced protein(s), it is sufficient to cause the release of cytochrome c from the mitochondria, activation of the apoptosome, caspases, and cell death. What is most surprising is that this occurs in cells bearing CrispR-induced mutations in both *Bax* and *Bak1*, and in which the corresponding proteins are undetectable by western blot.

How can these observations be explained? One possibility is that there is some residual BAX or BAK1, that is able to accumulate over several days until it reaches a level sufficient to allow release of cytochrome c. Reasons for discounting this possibility are firstly, that neither BAX nor BAK1 could be detected by western blot in the double-mutant cells at any stage, even after 6 days treatment with Dex; secondly, the monoclonal antibodies against BAX and BAK1 were directed at amino-terminal regions, so any remaining protein would have its antibody binding epitopes preserved; thirdly, if residual BAX or BAK1 were present, deleting BCL2, BCLXL and MCL-1 would have greatly increased the sensitivity of the cells to Dex, but we did not see this; and fourthly, mutation of *Bax* and *Bak1* had similar effects in an independent lymphoid line.

Another explanation could be that in addition to BAX and BAK1, BIM could activate another pro-apoptotic BCL2 family member, such as BOK or BID.

However, BOK was not detectable in the WEHI7 or the p53^−/−^ T lymphoma lines when they were untreated, or even after 6 days of treatment with Dex. Furthermore, mutation of BID had no impact on Dex-induced killing in *Bax*^−/−^*Bak1*^−/−^ WEHI7 cells.

Another possibility is that BIM is able to convert an anti-apoptotic BCL2 family member such as BCLXL, MCL-1, or BCL2 itself into a pro-apoptotic protein. Zhao et al. reported that treatment of *Bax/Bak1* double KO mouse embryonic fibroblasts with a diterpenoid compound-induced BIM, which migrated to the mitochondria, altered the conformation of BCL2, and together they permeabilized the outer mitochondial membrane to cause release of cytochrome c^20^. However, we do not believe this is happening in our lymphoid cells, as mutation of genes for BCL2, BCLXL or MCL-1, alone or in combination, did not prevent Dex induced, BIM-dependent release of cytochrome c or their death.

Mizuta et al.^21^ reported a BAX/BAK1 independent mechanism of cytochrome c release that caused apoptosis of mouse embryo fibroblasts. However, this serine protease-dependent process could not be inhibited by anti-apoptotic BCL2 family members, whereas the Dex induced, BIM-dependent process we observed in the lymphoid cells is inhibitable by anti-apoptotic BCL2 family members.

The explanation that remains is that Dex induces expression of BIM, and also one or more other, yet to be identified, proteins. These proteins enable BIM to form pores in the outer mitochondrial membrane, allowing release of Cytc. Although we have not yet identified this protein or proteins, we have shown it is not BAX, BAK1, BOK, BID, PUMA, BMF, VDAC2, BCL2, BCLXL, MCL-1, APAF1, Caspase-9, or p53.

## Materials and methods

### Cell culture

WEHI7 cells were cultured in RPMI 1640 medium (Life Technologies) supplemented with 8% fetal bovine serum (FBS, Sigma Cat# F9423). p53^−/−^ thymic lymphoma cells are previously described^11^ and were cultured in DME KELSO medium (Dulbecco’s Modified Eagle’s medium modified by Dr. Anne Kelso) supplemented with 250 µM l-asparagine, 50 µM 2-mercaptoethanol, and 10% fetal bovine serum. Cells were cultured at 37 °C and 10% CO_2_. All cell lines used were routinely tested for mycoplasma contamination.

### CRISPR/Cas9 gene mutation and inducible expression

For CRISPR/Cas9 gene deletion, parental cells were infected with lentiviral constructs encoding Cas9 and mCherry, and a doxycycline-inducible single-guide RNA targeting early protein coding exons of the desired gene, and GFP. Following selection of transduced cells by sorting double-positive cells on a flow cytometer (FACS, Becton Dickinson), cells were treated with 1 μg/ml doxycycline (Sigma) to induce sgRNA expression. Independent single-cell clones lacking the targeted protein were confirmed by immunoblotting and/or sequencing. Sequences of all sgRNA constructs used in this study are given in Supplementary Table 1. For inducible gene expression, the doxycycline-inducible lentiviral vector pFTRE 3G rtTA GFP/human BIMs, kindly provided by Prof. David Huang (WEHI), was used to infect cells, and clonal lines were obtained by sorting single GFP-positive cells, and inducible expression was confirmed by western analysis.

### Antibodies and reagents

Antibodies used were to ACTIN (AC-15, Sigma #A1978), MCL-1 (Rockland #600-401-394S), BAX (N-20 Santa Cruz Biotechnology #sc-493), BAK1 (aa23-38, #B5897 Sigma), BCL-2 (#610539, BD Biosciences), Cytochrome c (#556433, BD Biosciences), VDAC2 (M.T. Ryan, Monash University), Cleaved CASP3 (#9661, Cell Signaling Technology), Cleaved CASP-9 (#9509, Cell Signaling Technology), PUMA (#ab9645, Abcam), VDAC1(Abcam, ab15895), Rabbit polyclonal antibodies raised against amino acids 19–32 of mBOK (gift from Francine Ke, WEHI^22^), BID (BD Biosciences, #559681), BCLXL (#2764, Cell Signaling Technology), BIM, APAF1, BMF, and CASP9 are from in house (L. O’Reilly, WEHI).

BH3-mimetic ABT737 and venetoclax (ABT199) were obtained from Selleck (Houston, TX). The pan-caspase inhibitor Q-VD-OPH was purchased from MP Biomedicals Cat#03OPH109. IDN-6556 was purchased from MedChem Express LLC. Necrostatin-1 (Nec-1) was synthesized by TetraLogic Pharmaceuticals. Doxycycline and dexamethasone were purchased from Sigma-Aldrich.

### Western blot analysis of cell lysates and cytosolic fractions

For the detection of cytochrome c released into the cytosol, cells were permeabilized for 10 min on ice with 0.025% w/v digitonin in 20 mM HEPES (pH 7.5), 100 mM KCl, 2.5 mM MgCl2, and 100 mM sucrose supplemented with complete protease inhibitors (complete protease inhibitor cocktail tablets, Roche). After centrifugation (13,000 × *g*, 4 °C) for 5 min, aliquots of the supernatant (cytosolic fraction) were analyzed by western blotting. To produce whole-cell lysates, cells were treated with RIPA buffer (50 mM Tris-HCl, 0.1% SDS, 1% Nonidet P-40, 0.5% deoxycholate and 150 mM NaCl, pH 8.0) complemented with protease inhibitors.

Lysates of whole cells or cytosolic fraction in SDS sample buffer were electrophoresed through Tris-glycine gels (BioRad) and transferred to PVDF/nitrocellulose membrane. Membranes were then incubated with blocking buffer for 1 h at room temperature, followed by specific antibodies for 16 h at 4 °C. Binding of HRP-conjugated secondary antibodies was subsequently visualized on the ChemiDoc Touch Imaging System (Biorad) using the Luminata Forte enhanced chemiluminescence reagent (Millipore).

### Cell death assays

To assess integrity of the plasma membrane, cells were harvested and stained with 1 μg/ml of propidium iodide (PI). Samples were analyzed with FACSCalibur flow cytometers (BD Biosciences) and the acquired data analyzed with FlowJo software. Dead cells were defined as those that were stained by PI (PI^+^).

### Soft-agar assays

Cells were counted and mixed with growth media containing 0.35% agarose and then plated on top of a solidified layer of 0.7% agarose in 6-well plates and maintained for 2 weeks. Pictures were taken by using Bio-Rad Gel Doc System (Bio-Rad, Mississauga, Canada) and colonies were counted by the ImageJ software or visual inspection. The circularity function was set to 0.2 to 1.00, with the pixel size set to 0.1–Infinity. The cloning efficiency was calculated by dividing the number of colonies by the number of cells initially plated in each well and expressed as percentages with a logarithmic scale.

## Supplementary information

Supplementary Figure 1

Supplementary Figure 2

Supplementary Figure 3

Supplementary Figure 4

Supplementary Figure 5

Supplementary Figure 6

Supplementary Figure 7

Supplementary Figure 8

Supplementary Figure 9

Supplementary Figure 10

Supplementary Table 1

Supplementary Figures Legends
