# Supplementary figures and images for: Glucocorticoids can induce BIM to trigger apoptosis in the absence of BAX and BAK1
Source: Cell Death Dis. 2020 Jun 8;11(6):442. doi: 10.1038/s41419-020-2599-5 (PMC7280233; doi:10.1038/s41419-020-2599-5)

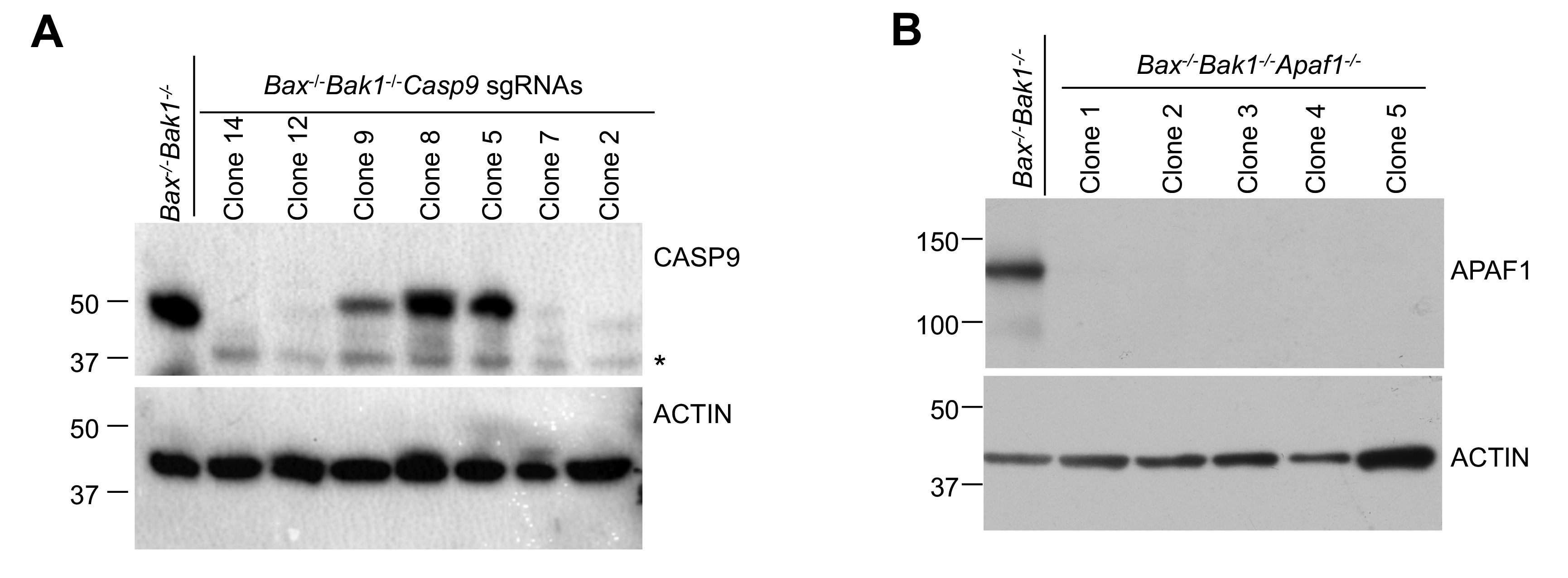

Supplement: Supplementary file 1 — Supplementary Figure 1 [file 41419_2020_2599_MOESM1_ESM.png]

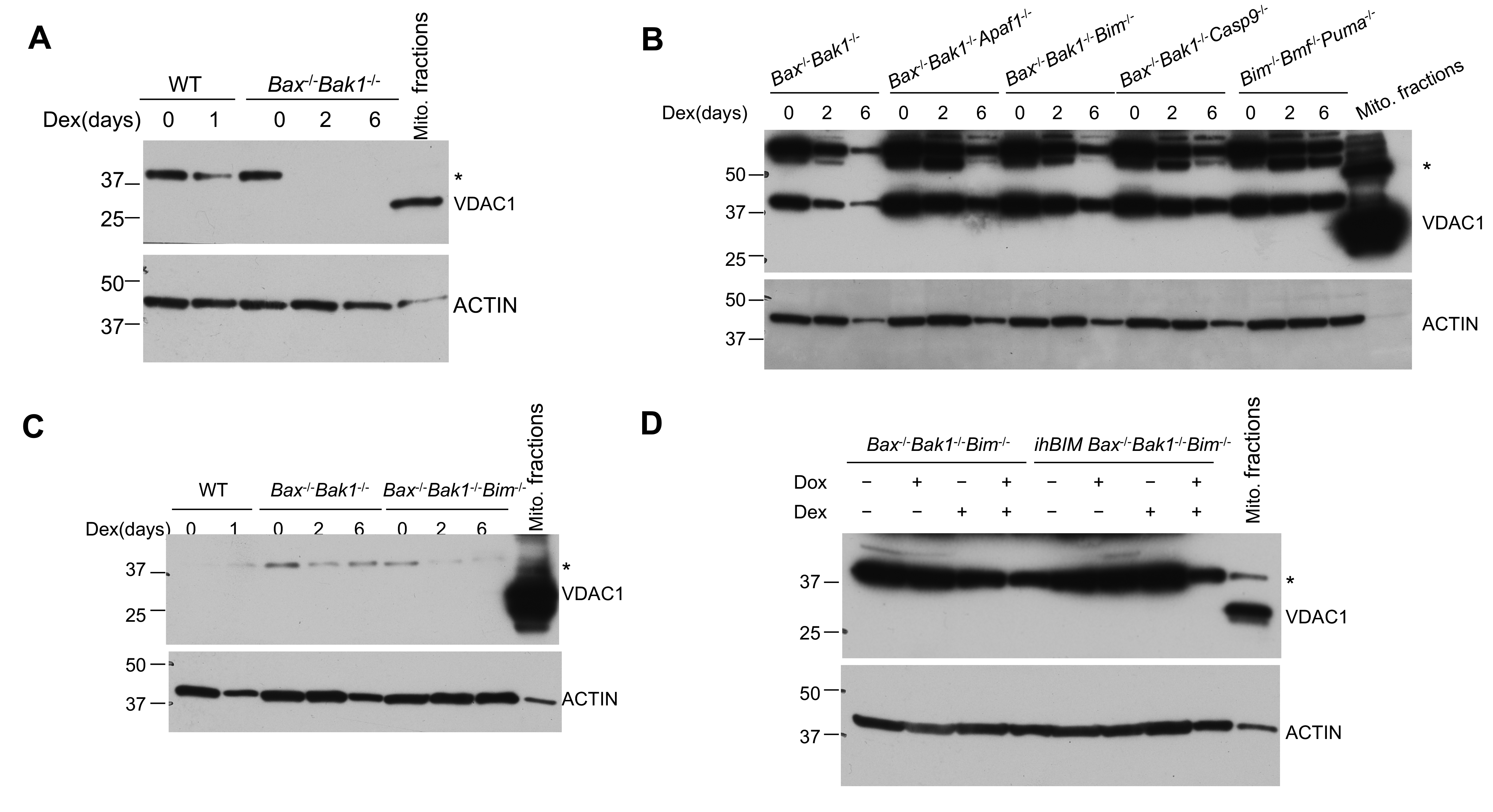

Supplement: Supplementary file 2 — Supplementary Figure 2 [file 41419_2020_2599_MOESM2_ESM.png]

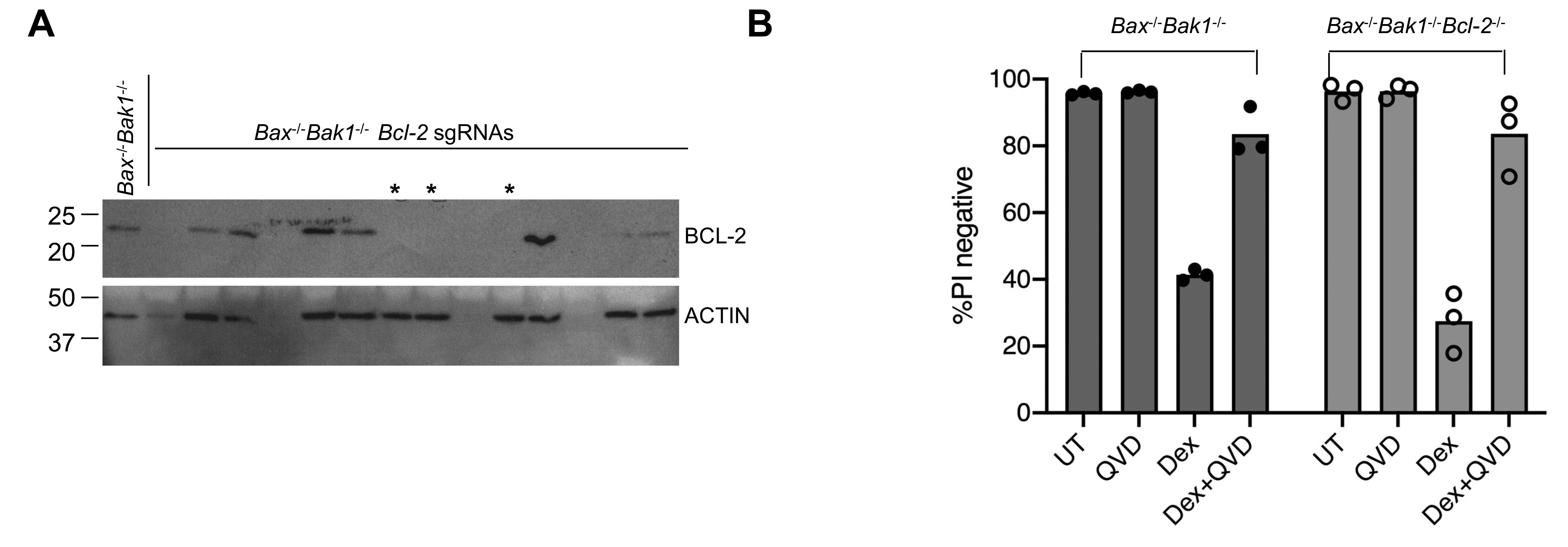

Supplement: Supplementary file 3 — Supplementary Figure 3 [file 41419_2020_2599_MOESM3_ESM.png]

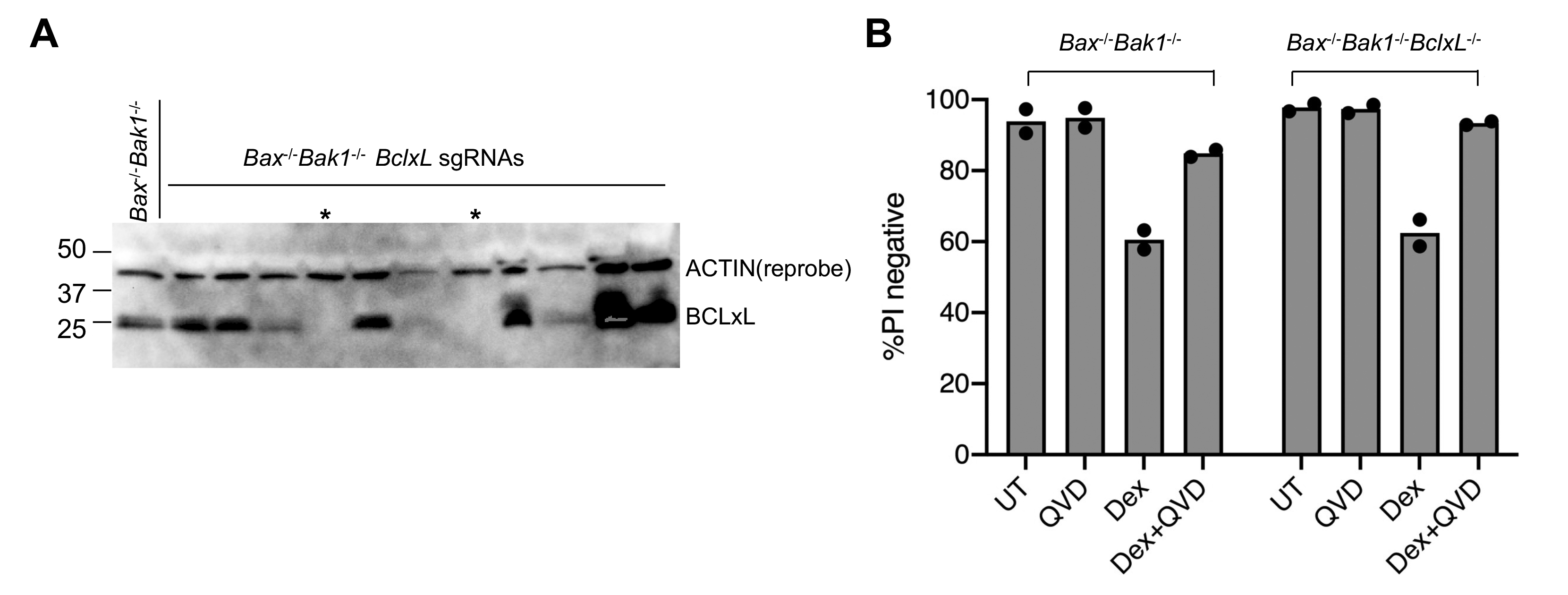

Supplement: Supplementary file 4 — Supplementary Figure 4 [file 41419_2020_2599_MOESM4_ESM.png]

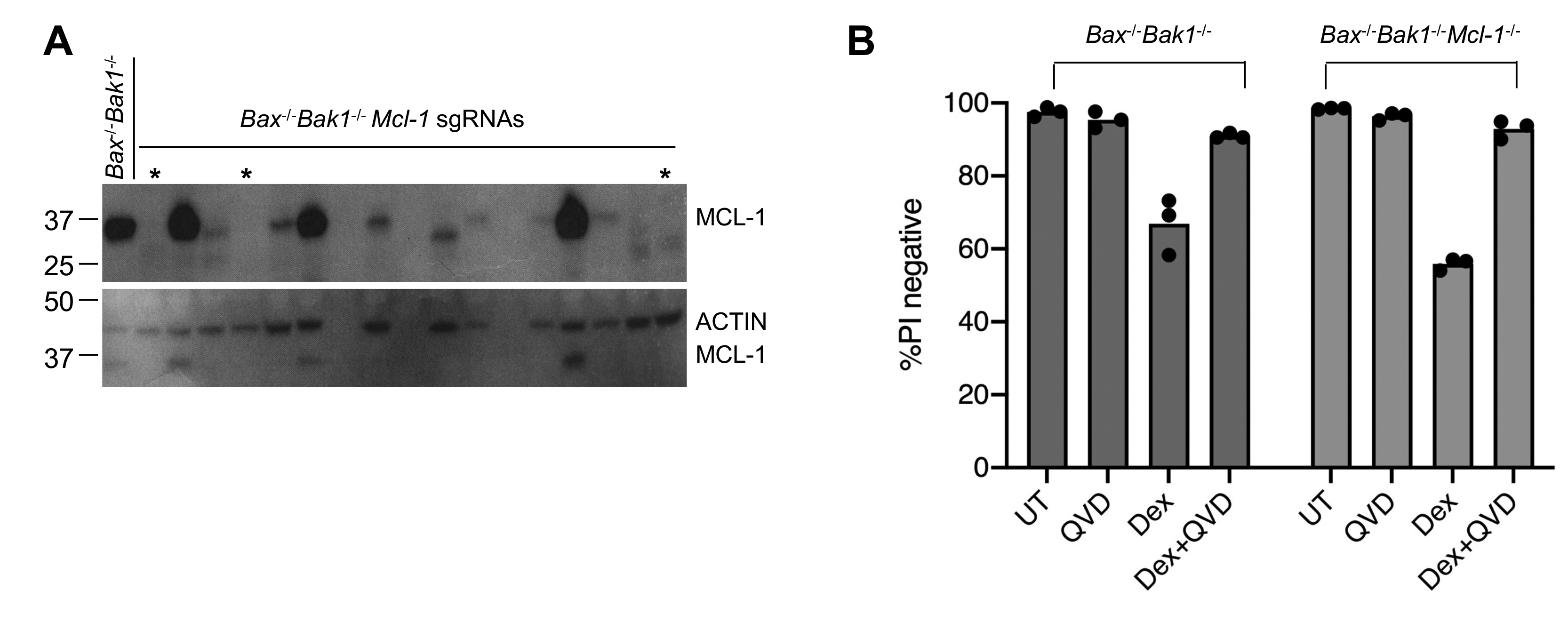

Supplement: Supplementary file 5 — Supplementary Figure 5 [file 41419_2020_2599_MOESM5_ESM.png]

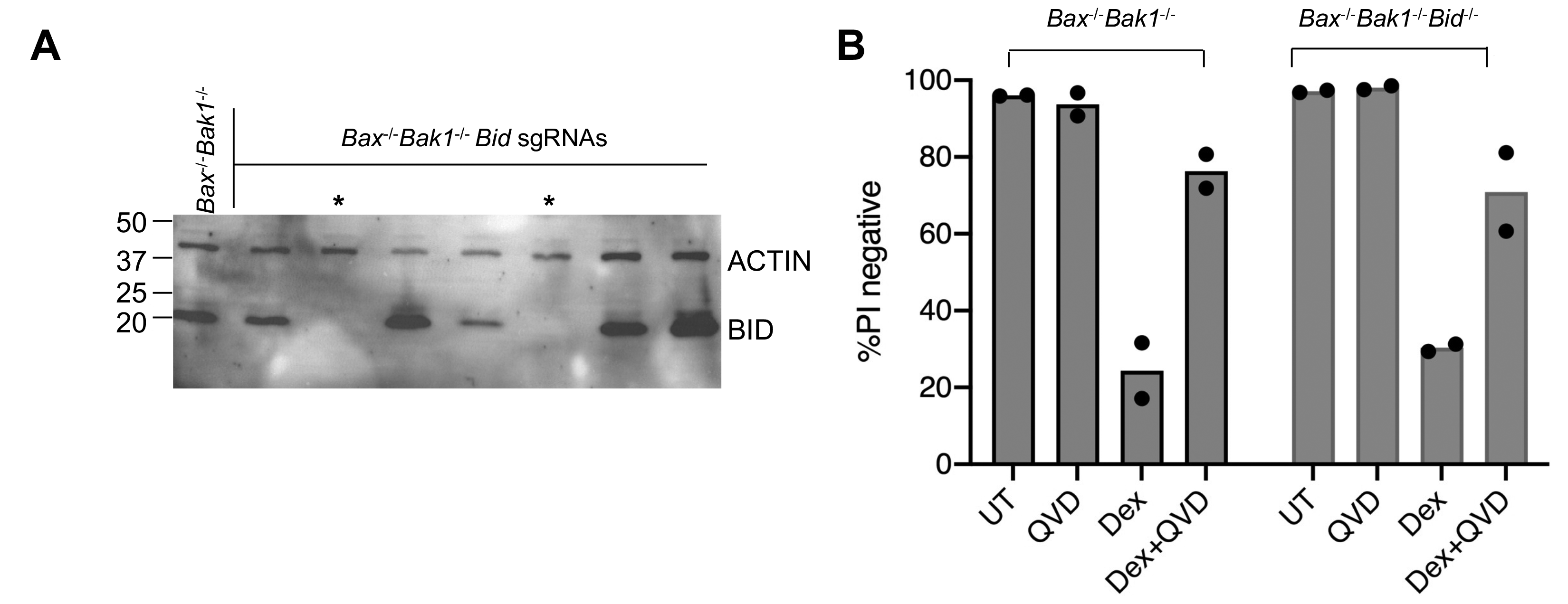

Supplement: Supplementary file 6 — Supplementary Figure 6 [file 41419_2020_2599_MOESM6_ESM.png]

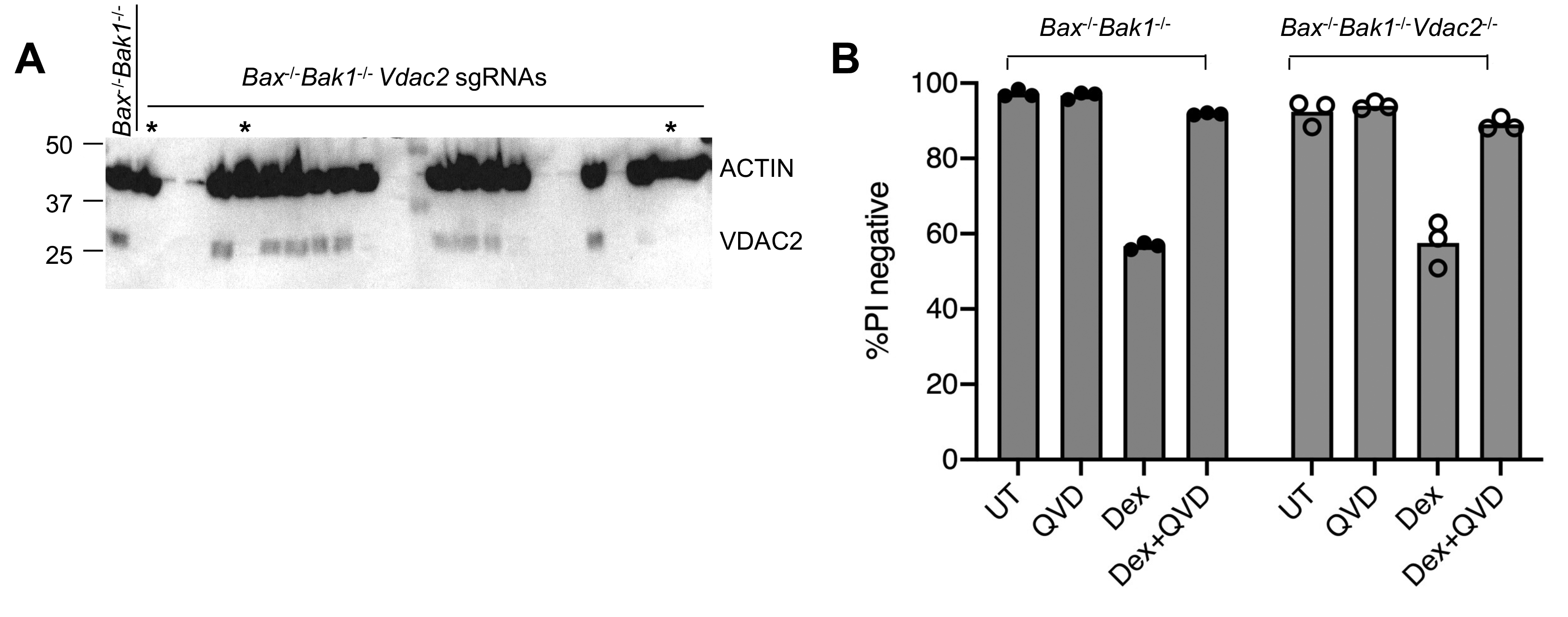

Supplement: Supplementary file 7 — Supplementary Figure 7 [file 41419_2020_2599_MOESM7_ESM.png]

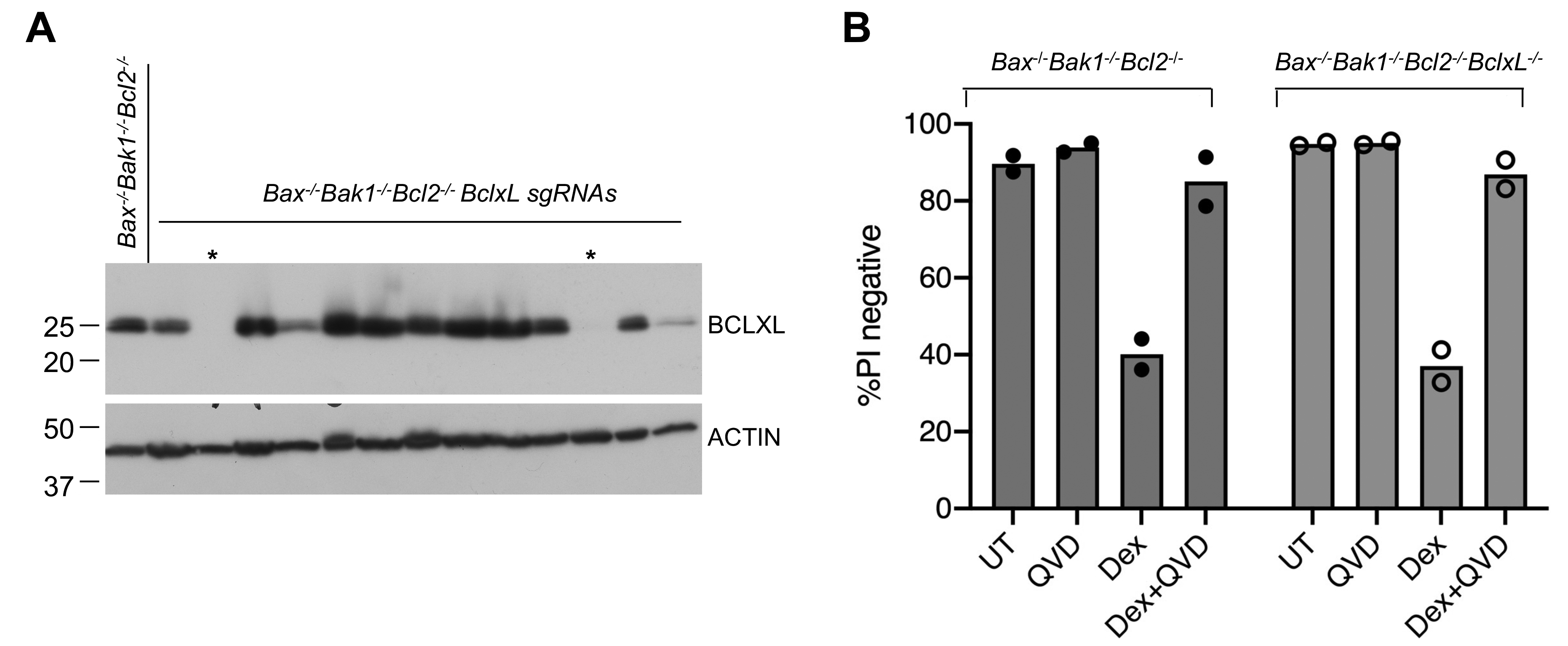

Supplement: Supplementary file 8 — Supplementary Figure 8 [file 41419_2020_2599_MOESM8_ESM.png]

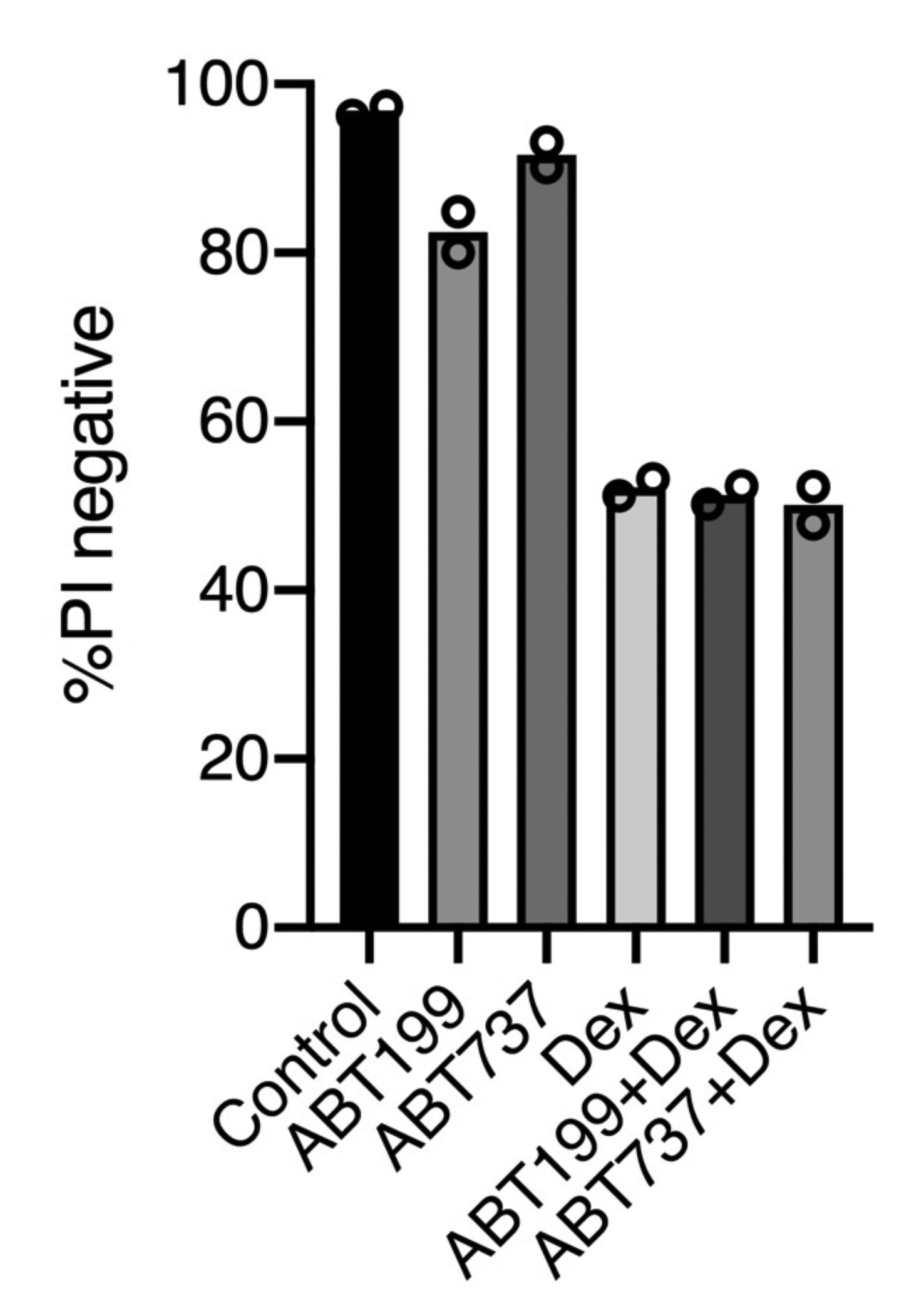

Supplement: Supplementary file 9 — Supplementary Figure 9 [file 41419_2020_2599_MOESM9_ESM.png]

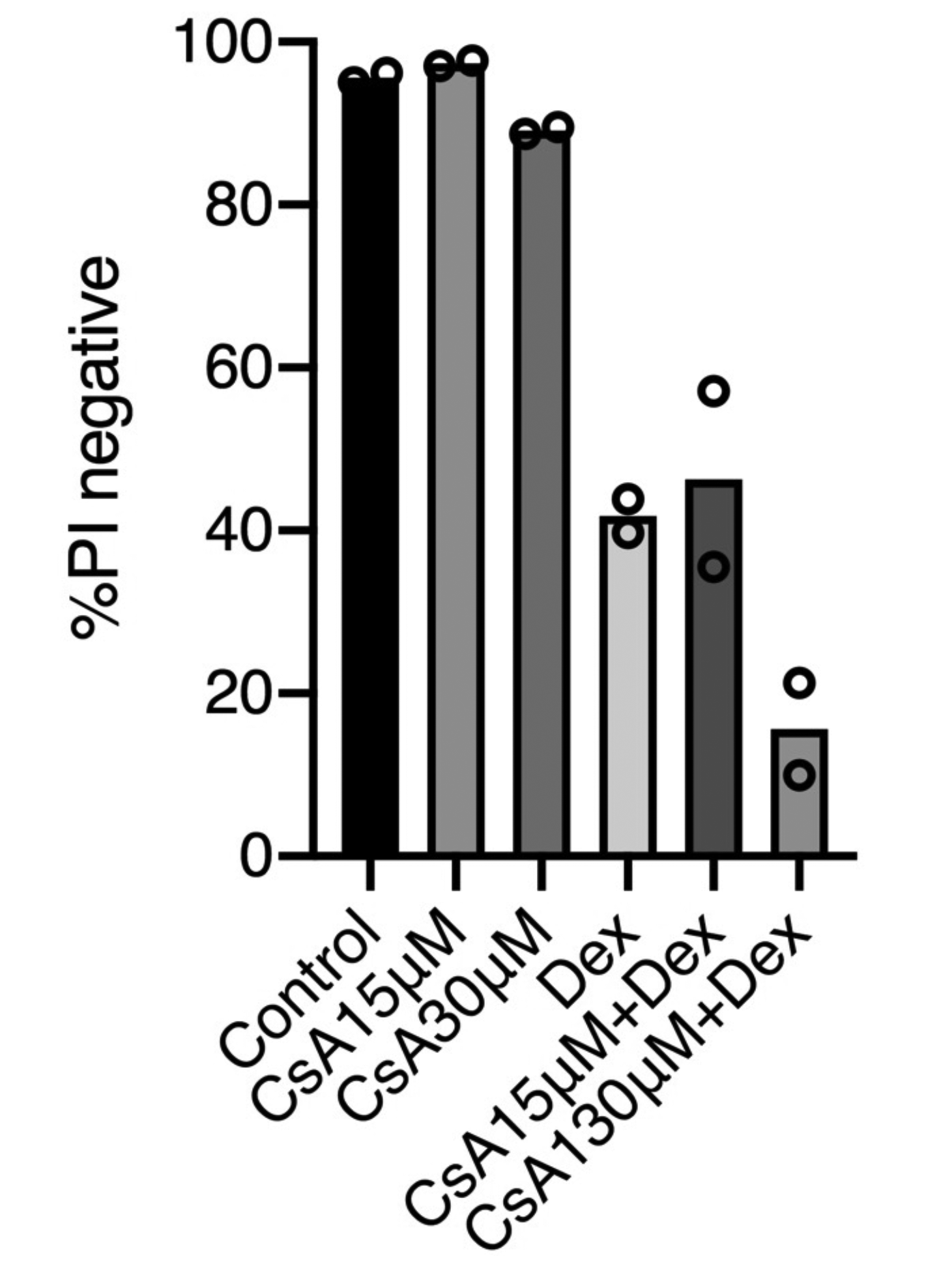

Supplement: Supplementary file 10 — Supplementary Figure 10 [file 41419_2020_2599_MOESM10_ESM.png]
