# Supplementary material for: Glucocorticoids can induce BIM to trigger apoptosis in the absence of BAX and BAK1
Source: Cell Death Dis. 2020 Jun 8;11(6):442. doi: 10.1038/s41419-020-2599-5 (PMC7280233; doi:10.1038/s41419-020-2599-5)
Supplement: Supplementary file 11 — Supplementary Table 1 [file 41419_2020_2599_MOESM11_ESM.docx]

Supplementary Table 1

Sequence of single guide RNA (sgRNA) used in this study.

| *Gene* | mouse sgRNA sequence |
| --- | --- |
| *BclxL* | GCTCTGATACGCGGTCCCT |
| *BclxL* | CGGATAGCCCGGCCGTGAA |
| *Mcl-1* | TGTAAGGACGAAACGGGACTGG |
| *Mcl-1* | AAGATGGCGTAACAAACTGGGG |
| *Caspase9* | CGACTTGCAGCTCGCTCACT |
| *Caspase9* | TTCACTCTTGCAAAGCGGT |
| *Apaf1* | CGAAAATAACCGGCCTCTG |
| *Apaf1* | ACGTGATTTTCCCAATCGC |
| *Bcl2* | CATCTCTGCGAAGTCACGA |
| *Bcl2* | CCGGTGCACAGCGGGCATT |
| *Bmf* | GAAGAGCTGGAGTCGACTG |
| *Bmf* | CTTGGTGCCCACACTCGAT |
| *Puma* | CCAGAGGCCCGCGCCCGG |
| *Puma* | GGGCAGGCCGGGCTCGCAA |
| *Bid* | GGTCCATCTCATCGCCTATT |
| *Bim* | GCACAGGAGCTGCGGCGGAT |
| *Vdac2* | GTGGAACACCGATAACACTC |
| *Bax* | AGTTTCATCCAGGATCGAGC |
| *Bak1* | TCATCGCAGCCCACCTTCGG |
