# Supplementary material for: Glucocorticoids can induce BIM to trigger apoptosis in the absence of BAX and BAK1
Source: Cell Death Dis. 2020 Jun 8;11(6):442. doi: 10.1038/s41419-020-2599-5 (PMC7280233; doi:10.1038/s41419-020-2599-5)
Supplement: Supplementary file 12 — Supplementary Figures Legends [file 41419_2020_2599_MOESM12_ESM.docx]

**Supplementary Figure Legends**

**Glucocorticoids can induce BIM to trigger apoptosis in the absence of BAX and BAK1**

Li Dong^1,2^ and David L Vaux^1,2*^

**Supplementary Figure S1.** *Generation of Bax^-/-^Bak1^-/-^ WEHI7 clones mutant for Caspase-9 and APAF1.*

**A.** *Bax*^-/-^*Bak1*^-/-^ WEHI7 cells expressing Cas9 were transduced with sgRNAs targeting *Caspase-9*. Following treatment with doxycycline to induce sgRNA expression, clones were isolated, and tested for absence of Caspase-9 by western blotting. Clones 2, 7, 12 and 14 were used in the analysis of cell death as shown in Figure 2B.

**B.** Whole cell lysates from *Bax^-/-^Bak1^-/-^* and 5 independent *Bax^-/-^Bak1^-/-^Apaf1^-/-^* cell clones were subjected to western blot analysis to detect APAF1 protein. These clones were used in the experiments shown in Figure 2B and 2C.

**Supplementary Figure S2.** *Assessing purity of the cytosolic fractions.*

**A.** Aliquots of the same cytosol samples shown in Fig. 2D were subjected to western blot analysis, probing with an antibody specific for the 31 kD mitochondrial protein VDAC1. A mitochondrial extract sample was used as a positive control. * unknown band.

**B.** Aliquots of the same cytosol samples shown in Fig 5C were subjected to western blot analysis, probing with an antibody specific for the 31 kD mitochondrial protein VDAC1. A mitochondrial extract sample was used as a positive control. * unknown band.

**C.** Aliquots of the same cytosol samples shown in Fig 5D were subjected to western blot analysis, probing with an antibody specific for the 31 kD mitochondrial protein VDAC1. A mitochondrial extract sample was used as a positive control. * unknown band.

**D.** Aliquots of the same cytosol samples shown in Fig 6D were subjected to western blot analysis, probing with an antibody specific for the 31 kD mitochondrial protein VDAC1. A mitochondrial extract sample was used as a positive control. * unknown band.

In each experiment, the mitochondrial protein VDAC1 was not detected in the cytosol extracts, showing that they were not contaminated with mitochondria, and that Cytochrome-c present in these fractions had been released from the mitochondria.

**Supplementary Figure S3.** *Generation of Bax^-/-^Bak1^-/-^ clones lacking BCL2, and testing for their resistance to Dex induced killing.*

**A.** *Bax*^-/-^*Bak1*^-/-^ WEHI7 cells bearing a Cas9 expression construct were transduced with sgRNAs targeting *Bcl2*. Following treatment with doxycycline to induce sgRNA expression, clones were isolated, and absence of BCL2 was determined by western blotting. The clones labelled * were used in Fig. S3B.

**B.** Three independent clones of *Bax*^-/-^*Bak1*^-/-^ and *Bax*^-/-^*Bak1*^-/-^ *Bcl2*^-/-^ WEHI7 cells were treated with 1 µM Dex for 6 days, and cell viability assessed by PI exclusion. Presence or absence of BCL2 had no impact on killing of *Bax*^-/-^*Bak1*^-/-^ by Dex.

**Supplementary Figure S4.** *Generation of Bax^-/-^Bak1^-/-^ clones lacking BCLXL and testing for their resistance to Dex induced killing.*

**A.** *Bax*^-/-^*Bak1*^-/-^ WEHI7 cells bearing a Cas9 expression construct were transduced with sgRNAs targeting *BclxL*. Following treatment with doxycycline to induce sgRNA expression, clones were isolated and absence of BCLXL was determined by western blotting. The clones labelled * were used in Fig. S3B.

**B.** Two independent clones of *Bax*^-/-^*Bak1*^-/-^ and *Bax*^-/-^*Bak1*^-/-^ *Bclxl*^-/-^ WEHI7 cells were treated with 1 µM Dex for 5 days, and cell viability assessed by PI exclusion. Presence or absence of BCLXL had no impact on killing of *Bax*^-/-^*Bak1*^-/-^ by Dex.

**Supplementary Figure S5.** *Generation of Bax^-/-^Bak1^-/-^ clones lacking MCL-1 and testing for their resistance to Dex induced killing.*

**A.** *Bax*^-/-^*Bak1*^-/-^ WEHI7 cells bearing a Cas9 expression construct were transduced with sgRNAs targeting *Mcl-1*. Following treatment with doxycycline to induce sgRNA expression, clones were isolated, and absence of MCL-1 was determined by western blotting. The clones labelled * were used in Fig. S5B.

**B.** Three independent clones of *Bax*^-/-^*Bak1*^-/-^ and *Bax*^-/-^*Bak1*^-/-^ *Mcl-1*^-/-^ WEHI7 cells were treated with 1 µM Dex for 5 days, and cell viability assessed by PI exclusion. Presence or absence of MCL-1 had no impact on killing of *Bax*^-/-^*Bak1*^-/-^ by Dex.

**Supplementary Figure S6.** *Generation of Bax^-/-^Bak1^-/-^ clones lacking BID and testing for their resistance to Dex induced killing.*

**A.** *Bax*^-/-^*Bak1*^-/-^ WEHI7 cells bearing a Cas9 expression construct were transduced with sgRNAs targeting *Bid*. Following treatment with doxycycline to induce sgRNA expression, clones were isolated and absence of BCLXL was determined by western blotting. The clones labelled * were used in Fig. S6B.

**B.** Two independent clones of *Bax*^-/-^*Bak1*^-/-^ and *Bax*^-/-^*Bak1*^-/-^ *Bid*^-/-^ WEHI7 cells were treated with 1 µM Dex for 7 days, and cell viability assessed by PI exclusion. Presence or absence of BID had no impact on killing of *Bax*^-/-^*Bak1*^-/-^ by Dex.

**Supplementary Figure S7.** *Generation of Bax^-/-^Bak1^-/-^ clones lacking VDAC2 and testing for their resistance to Dex induced killing.*

**A.** *Bax*^-/-^*Bak1*^-/-^ WEHI7 cells bearing a Cas9 expression construct were transduced with sgRNAs targeting *Vdac2*. Following treatment with doxycycline to induce sgRNA expression, clones were isolated and absence of VDAC2 was determined by western blotting. Three of the clones labelled * were used in Fig. S7B.

**B.** Three independent clones of *Bax*^-/-^*Bak1*^-/-^ and *Bax*^-/-^*Bak1*^-/-^ *Vdac2*^-/-^ WEHI7 cells were treated with 1 µM Dex for 5 days, and cell viability assessed by PI exclusion. Presence or absence of VDAC2 had no impact on killing of *Bax*^-/-^*Bak1*^-/-^ by Dex.

**Supplementary figure S8.** *Generation of Bax^-/-^Bak1^-/-^ clones lacking both BCL2 and BCLXL, and testing for their resistance to Dex induced killing.*

**A.** *Bax*^-/-^*Bak1*^-/-^*Bcl-2*^-/-^ WEHI7 cells were transduced with sgRNAs targeting *Bclxl*. Following treatment with doxycycline to induce sgRNA expression, clones were isolated, and absence of BCLXL was determined by western blotting. The clones labelled * were used in Fig. S8B.

**B.** Two independent clones of *Bax*^-/-^*Bak1*^-/-^ *Bcl-2*^-/-^ and *Bax*^-/-^*Bak1*^-/-^ *Bcl-2*^-/-^ *Bcl-xL*^-/-^ WEHI7 cells were treated with 1 µM Dex for 6 days, and cell viability assessed by PI exclusion. Presence or absence of BCLXL had no impact on killing of *Bax*^-/-^*Bak1*^-/-^ *Bcl-2*^-/-^ cells by Dex.

**Supplementary Figure S9.** *ABT737 or ABT199 did not affect Dex induced Bax/Bak1 independent killing*

Two independent clones of *Bax^-/-^Bak1^-/-^* WEHI7 cells were treated with 1 µM Dex and/or 1.1 µM ABT199 or 3.3 µM ABT737 for 5 days. Cells were harvested, resuspended in PBS containing propidium iodide (PI) and analyzed by flow cytometry. Consistent with BCL-2 and BCLXL playing no role in the death of *Bax^-/-^Bak1^-/-^* WEHI7 induced by Dex, the BCL-2 specific inhibitor ABT199 and the BCL2/BCLXL dual inhibitor ABT737 had no impact on death induced by Dex.

**Supplementary Figure S10.** *MPTP inhibitor cyclosporine A (CsA) could not prevent Dex induced BAX/BAK1 independent killing*

Two independent clones of *Bax^-/-^Bak1^-/-^* WEHI7 cells were treated with 1 μM Dex and/or the MPTP inhibitor cyclosporine A (CsA) at the indicated concentrations for 6 days. Cells were harvested, resuspended in PBS containing PI and analyzed by flow cytometry. The MPTP inhibitor CsA did not reduce killing of *Bax^-/-^Bak1^-/-^* cells by Dex.
